# Enhanced Molecular Networking Shows *Microbacterium* sp. V1 as a Factory of Antioxidant Proline-Rich Peptides

**DOI:** 10.3390/md21040256

**Published:** 2023-04-21

**Authors:** Giovanni Andrea Vitale, Silvia Scarpato, Alfonso Mangoni, Maria Valeria D’Auria, Gerardo Della Sala, Donatella de Pascale

**Affiliations:** 1 Department of Eco-Sustainable Marine Biotechnology, Stazione Zoologica Anton Dohrn, Via A.F. Acton, Molosiglio, 80133 Naples, Italy; giovanniandrea.vitale@szn.it; 2 Department of Pharmacy, University of Naples “Federico II”, 80131 Naples, Italy; silvia.scarpato@unina.it (S.S.); alfonso.mangoni@unina.it (A.M.); madauria@unina.it (M.V.D.); 3 GEOMAR Centre for Marine Biotechnology (GEOMAR-Biotech), Research Unit Marine Natural Products Chemistry, GEOMAR Helmholtz Centre for Ocean Research Kiel, Am Kiel-Kanal 44, 24106 Kiel, Germany

**Keywords:** marine bacteria, OSMAC, molecular networking, proline-rich peptides, structure elucidation, antioxidant, bioactive peptides

## Abstract

Two linear proline-rich peptides (**1**–**2**), bearing an N-terminal pyroglutamate, were isolated from the marine bacterium *Microbacterium* sp. V1, associated with the marine sponge *Petrosia ficiformis*, collected in the volcanic CO_2_ vents in Ischia Island (South Italy). Peptide production was triggered at low temperature following the one strain many compounds (OSMAC) method. Both peptides were detected together with other peptides (**3**–**8**) via an integrated, untargeted MS/MS-based molecular networking and cheminformatic approach. The planar structure of the peptides was determined by extensive 1D and 2D NMR and HR-MS analysis, and the stereochemistry of the aminoacyl residues was inferred by Marfey’s analysis. Peptides **1**–**8** are likely to arise from *Microbacterium* V1 tailor-made proteolysis of tryptone. Peptides **1** and **2** were shown to display antioxidant properties in the ferric-reducing antioxidant power (FRAP) assay.

## 1. Introduction

The genus *Microbacterium* belongs to the family *Microbacteriaceae* within the *Actinomycetota* phylum (formerly *Actinobacteria*). More than 120 species of this genus have been isolated from terrestrial and marine environments [1]. Common features of this genus are a resistance to heavy metals, the ability to increase metal ion mobility, and the capacity of reducing several metal ions, such as Cr^6+^. Indeed, numerous *Microbacterium* species have been retrieved from contaminated sites [2,3]. Concomitantly, several other species were found to boost plant growth, by possessing specific plant growth-promoting (PGP) traits [4]. These features made *Microbacterium* a suitable candidate for bioremediation purposes and, therefore, this genus has been studied to be exploited for soil decontamination via the phytoextraction process.

Over the last few years there has been a growing interest in lesser-studied *Actinomycetes* members, the so-called “Rare *Actinomycetes*”, including *Microbacteriaceae* [5]. 

In this context, a survey analysis of 70 *Microbacterium* genomes showed that their genomes are much shorter than those of other *Actinomycetota* genera, such as *Streptomyces*, which are considered the most prolific bacteria in the field of natural products (NPs) [6]. However, *Microbacterium* genomes present on average a very small core, addressing primary functions, while possessing instead a high percentage of accessory genes, therefore representing a valuable source of secondary metabolites (SMs) [7].

Another pool of potentially bioactive molecules besides SMs is represented by the so-called bioactive peptides (BAPs) [8,9]. These small sequences are hidden within common food proteins where they are inactive, and can be released as a consequence of an in vitro enzymatic or microbial-mediated hydrolysis. Numerous BAPs are reported to feature angiotensin-converting enzyme (ACE)-inhibitory, antimicrobial, immunomodulating, and antioxidant activities [10,11,12,13,14,15], as some of them resemble the structure of human endogenous peptides and can interact with the same receptors [15,16,17]. As a result, besides their nutritional value, proteins derived from different sources are being extensively studied for the positive impact they can have on human health in terms of their BAPs supply. The most studied bacterial genus for BAPs production is certainly *Lactobacillus*, with several species from this genus being currently used in various food industrial applications [18]. *Lactobacillus* proteolytic activity is largely due to its auxotrophy for certain amino acids, which forced the bacterium to develop efficient proteolytic machinery to overcome this issue. Micro-organisms represent the most promising source of BAPs. In particular, strains of the genus *Lactobacillus* are currently industrially employed for producing marketed fermented products, including probiotics and functional foods, where fermentation confers improved healthy features due to the BAP’s functionalization. However, challenges in this field are mainly represented by the identification and isolation of new BAPs within complex matrices, and the efficient upscaling for industrial production [13,15].

In this regard, the major drawback in natural product discovery, mainly observed when a classical bioactivity-driven approach is employed, is the “rediscovery” of known compounds, while new metabolites showing no activity in the selected test risk being overlooked. Today, the field of natural products is being totally revolutionized by numerous inventions related to OMIC technologies, including genomics and metabolomics [19,20,21,22]. Significant advances in metabolomics include the upstream chemical dereplication of metabolites, to guide the time-consuming purification process towards targeted isolation of novel natural products. Major improvements are certainly ascribable to the Global Natural Product Social Molecular Networking (GNPS) platform, a metabolomic ecosystem where query tandem mass (MS/MS) spectra are clustered based on peak pattern similarity and annotated through comparison with spectral libraries. Here users can process, annotate, and share MS/MS data of samples from natural sources, thereby significantly boosting the dereplication of unknown molecules [23]. Along with molecular networking, numerous tools aimed at improving the dereplication process have been set up for the prediction of molecular fingerprints, chemical class assignment, and in silico structure prediction of unknown metabolites [24,25].

This study delved into the metabolic potential of the marine *Microbacterium* sp. V1, isolated from the Mediterranean sponge *Petrosia ficiformis*. Using the “One Strain Many compounds“ (OSMAC) approach [26], we explored different stimuli to fuel bacterial metabolic pathways leading to the putative production of novel natural products. Untargeted metabolic profiling of eight OSMAC conditions revealed several metabolites almost exclusively present in one culture condition, i.e., lysogeny broth (LB) medium at 15 °C. Molecular networking analysis of liquid chromatography–high-resolution tandem mass spectrometry (LC-HRMS/MS) data along with cheminformatic class prediction, led to the annotation of three unique molecular clusters as peptides, featuring proline redundancy in their sequences. Isolation and stereo-structural elucidation of the two most abundant proline-rich peptides (**1**–**2**) showed them to bear an uncommon N-terminal pyroglutamate residue. These compounds, here reported for the first time, are likely to arise from *Microbacterium* V1 tailor-made proteolysis of tryptone and were shown to display antioxidant properties in the ferric-reducing antioxidant power (FRAP) assay. 

## 2. Results and Discussion

### 2.1. Bacterial Isolation from the Sponge Petrosia Ficiformis

Bacterium strain V1 *Microbacterium_sp* was isolated with PDA, from a marine sponge *Petrosia ficiformis* of white colouration phenotype (sample code: GMPFB1), collected in the acidified site Grotta del Mago (pH = ~7), Ischia Island (Naples, Italy). This peculiar area is considered a natural laboratory for studying ocean acidification; here, the presence of submerged CO_2_ vents causes a local drop in the pH [27]. The sponge *Petrosia ficiformis,* which is ubiquitously spread in the Mediterranean Sea, was collected from this acidified environment and used for microbial isolation at acidified pH (5–7) on Marine Broth (MB) plates, as we extensively described before for another sponge species collected in the same area [28].

Twelve morphologically different strains were isolated and identified at the genus level through 16S rRNA sequencing, belonging to the genera: *Pseudoalteromonas*, *Sphingobium*, *Microbacterium*, *Brevundimonas*, *Stenotrophomonas*.

The genus *Microbacterium* belongs to the family *Microbacteriaceae* within the *Actinomycetota* phylum and is commonly characterized by a high GC content.

It is well known that *Actinomycetota* represent the most prolific phyla in terms of natural products (NPs) production. To date, 29% of the overall NPs toolbox, and 60% of antibiotics included in clinical settings, come from *Actinomycetota*, and mainly from the genus *Streptomyces*, making them the most important micro-organisms in several fields, including biotechnology, agriculture, and pharmaceutics. However high efforts in the biotechnological exploitation of the genus *Streptomyces* resulted in high rediscovery rate in recent years, thus pushing the researchers to move towards less-studied families within *Actinomycetota*, also known as “Rare *Actinomycetota*”, including *Pseudonocardiaceae*, *Microbacteriaceae*, and *Micromonosporaceae* [5]. For this reason, the strain *Microbacterium* V1 was selected for further metabolomic studies.

### 2.2. OSMAC-Based Cultivation and Comparative Metabolic Profiling

The fast development of sequencing techniques, together with the increasing amount of deposited microbial genome sequences, revealed an inconsistency between the identified biosynthetic gene clusters (BGCs) and the number of metabolites concretely isolated in the laboratory. This is due to the limited stimulus provided in standard laboratory cultivations, which are often unable to activate certain BGCs and/or enzymatic functions. 

The One Strain Many Compounds Approach (OSMAC) approach is a cultivation-based strategy specifically conceived to activate the so-called “silent BGCs” by providing different stimuli in terms of cultivation parameters, including nutrient sources such as carbon, nitrogen and phosphorous, salt concentration, and temperature [28,29]. These variables become even more relevant in marine micro-organisms, considering the high variability of these parameters in their natural habitats [26]. 

In the present study, *Microbacterium* sp. V1 was cultivated in eight different conditions focusing our attention on two parameters: medium and temperature. Four media (LB, MB, ISP2, and SV2) and two temperatures (15 °C and 30 °C) were selected.

After five days, the cultures were centrifuged, and the supernatants and the pellets were extracted as reported in Section 3.3 to afford eight intracellular and eight extracellular extracts, which were dissolved in mass-grade MeOH and analyzed through LC-HRMS/MS.

**Figure 1:**
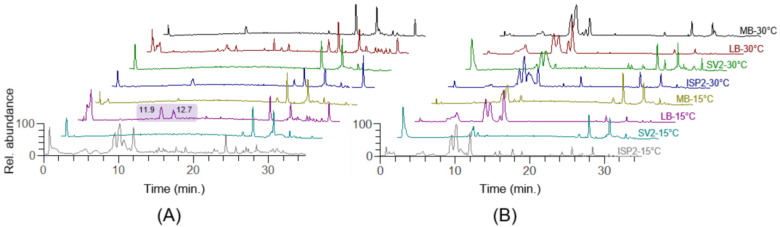
Overlay of LC-MS chromatograms of crude extracts from pellets (**A**) and supernatants (**B**) of *Microbacterium* sp. V1 cultivated in eight different growth conditions. The intracellular extract of *Microbacterium* sp. V1 cultivated in LB medium at 15 °C, was shown to contain higher amounts of the mid-polar compounds at *m*/*z* 635.34 and 534.29 (circled in pink), eluted at retention times 12.7 and 11.9 min, respectively, and later identified as **1** and **2**. LC-MS chromatograms are labeled with the culture medium abbreviation and the bacterial growth temperature.

Overlay of LC-MS chromatograms of the 16 crude extracts revealed some notable differences (Figure 1), especially concerning extracts obtained in LB medium at 15 °C and 30 °C. Indeed, while the LB extracellular extracts at different temperatures (red and pink profiles in Figure 1B) seem to display a similar profile, the LB intracellular extract obtained at 15 °C (pink profile in Figure 1A) showed two higher peaks in the mid-polar region of the chromatogram (11–13 min). A preliminary analysis of the isotope and fragmentation patterns disclosed two potentially new metabolites at *m*/*z* 635 (*R*_t_ = 12.7 min) and 534 (*R*_t_ = 11.9 min), bearing a peptide structure ([M + H]^+^ = *m*/*z* 635.3392, C_30_H_47_N_6_O_9_^+^; [M + H]^+^ = *m*/*z* 534.2915, C_26_H_40_N_5_O_7_^+^). These findings prompted us to perform the up-scaled cultivation of the bacterium in the optimal culture condition for purification purposes. Therefore, a 3 L cultivation in LB at 15 °C was set up and the bacterial pellet was afforded and extracted as described in Section 3.3. The crude extract was subjected to reversed-phase chromatography by using a vacuum chamber equipped with an SPE C-18 column and five fractions were eluted using different mixtures of H_2_O and MeOH, ranging from 100% H_2_O to 100% MeOH.

### 2.3. Molecular Network of Microbacterium sp. V1 Grown in LB Medium at 15 °C

The SPE fractions were dissolved in mass-grade MeOH and subjected to chemical profiling, to investigate in depth the metabolome of *Microbacterium* sp. V1 in the selected culture condition. All the fractions were analyzed through an LC-HRMS/MS data-dependent analysis (DDA) as described in Materials and Methods. Briefly, this method allowed acquiring simultaneously MS and MS/MS of the most abundant ions, for the entire duration of the chromatographic run. The .raw file obtained for each SPE fraction was processed by MZmine [30] for noise removal and data processing and alignment (Table S1), and the output files were submitted to the Global Natural Products Social Molecular Networking (GNPS) platform [23], to build a feature-based molecular network (FBMN). The FBMN thus generated was subjected to the MolNetEnhancer workflow to enhance MN annotation through integration of in silico library and dereplicator data and allow for automated classification of the molecular clusters through the ClassyFire algorithm [31].

**Figure 2:**
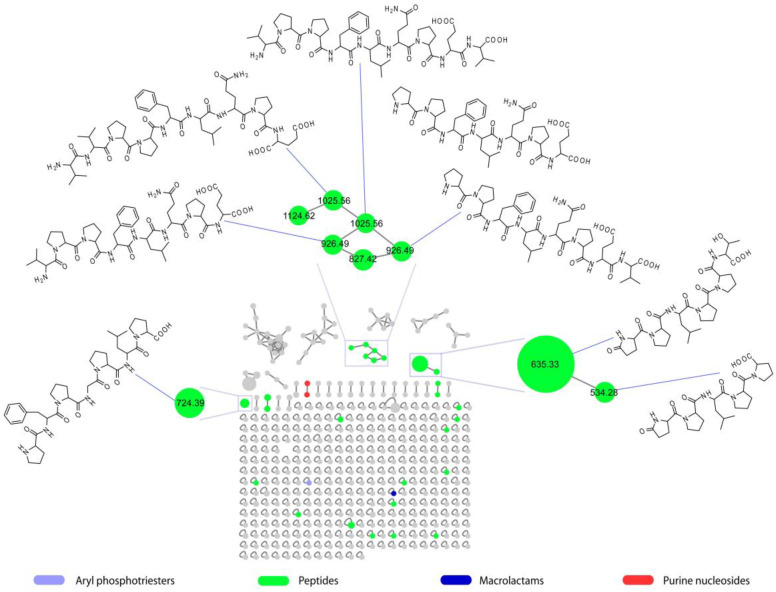
The molecular network (MN) of the SPE fractions from the crude extract of *Microbacterium* sp. V1 cultivated in LB medium at 15 °C. The MN was annotated by MolNetEnhancer. Herein, each node is colored based on its chemical classification as indicated in the color chart, while the node size is directly proportional to the precursor ion intensity. Structures of peptides discussed in this study are included in the MN and linked to the relevant nodes.

Peptides were the most represented molecular class in the MN (Figure 2), including 27 nodes that could not be associated with any known metabolite reported in the GNPS spectral database and, therefore, could indicate novel compounds. Eight of the nodes annotated as peptides were structurally elucidated by using high-resolution ESI-MS/MS and/or homo- and heteronuclear 2D NMR experiments and found to be proline-rich linear peptides (PRPs). 

### 2.4. Purification and Structure Elucidation of Proline-Rich Peptides 1 and 2 from Microbacterium sp. V1

**Figure 3:**
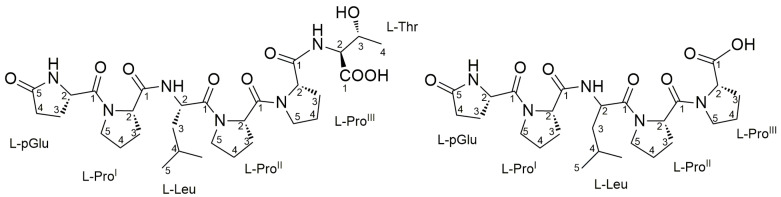
Structures of the proline-rich linear peptides **1** and **2**.

The two most abundant PRPs ([M + H]^+^ ions at *m*/*z* 635.34 and *m*/*z* 534.29, respectively) were mainly present in the SPE fraction eluted with 25% MeOH (fraction B). Therefore, fraction B was subjected to a single step of reversed-phase HPLC chromatography (see Section 3.5), to afford the pure compounds **1** and **2** (Figure 3).

The high-resolution ESI mass spectrum of **1** showed the [M + H]^+^ pseudomolecular ion at *m*/*z* 635.3392 (Figure S1), which indicated the molecular formula C_30_H_46_N_6_O_9_ with 11 unsaturations. The fragmentation spectrum of **1** suggested a linear hexapeptide structure, with the *b_5_* fragment arising from the neutral loss of a terminal threonine. Identification of the *b*-, *y*-, and *a*-type ion series in the MS/MS spectrum of **1** allowed for the prediction of the peptide sequence as X-X-Leu-Pro-Pro-Thr (Figure S2). The molecular formula was satisfied with the presence of one pyroglutamic acid and one further proline residues, thus accounting for the 11 degrees of unsaturation and completing the amino acid composition of **1**, later confirmed by NMR analysis. 

**Figure 4:**
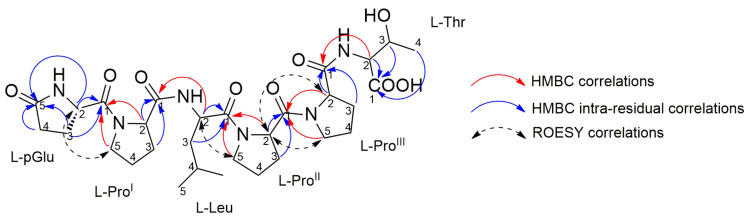
Diagnostic HMBC and ROESY correlations used to elucidate amino acid sequence in compound **1**. Correlations used to assign carbonyl ^13^C signals are noted with red arrows, intra-residual correlations are noted with blue arrows. ROESY correlations are noted with black arrows.

The structure of **1** was unambiguously determined through detailed analysis of a full set of homonuclear and heteronuclear two-dimensional NMR spectra (COSY, TOCSY, ROESY, HSQC, and HMBC) (Figures S3–S12). The ^1^H NMR spectrum showed six α-proton signals, as expected for a hexapeptide. Interpretation of cross peaks with the corresponding α-proton signals in the TOCSY spectrum allowed the identification of six independent spin systems. The assignment of each residue was achieved using the COSY and HSQC spectra. A band-selective HSQC experiment was also performed to improve resolution in the ^13^C dimension, enabling the proline residue signals to be distinguished. Further confirmation of the signal assignment of the different proline residues was achieved with the analysis of a band-selective HMBC experiment. The amino acid sequence in the peptide was determined from HMBC data. In addition to the standard HMBC experiment, a band-selective HMBC was performed allowing the discrimination of CO signals with close ^13^C chemical shifts, such as pGlu-C1 and Leu-C1. Key HMBC correlations to elucidate the amino acid sequence are reported in Figure 4. The structure was established to be a linear pGlu-Pro^I^-Leu-Pro^II^-Pro^III^-Thr, which was confirmed by the correlations observed during the ROESY NMR experiment (Table 1) and consistent with the structural prediction by MS/MS analysis.

The absolute configuration of the six amino acid residues of compound **1**, was clarified by an advanced Marfey’s methodology. A high-resolution Orbitrap MS instrument was used as a detector to analyze the reaction results, improving sensitivity and specificity. Compound **1** (100 μg) was subjected to total hydrolysis by treating it with 6 N HCl/AcOH (1:1) at 120 °C for 18 h. The derivatization of the resulting amino acid residues with the L enantiomer of Marfey’s reagent (1-fluoro-2-4-dinitrophenyl-5-L-alanine amide, or L-FDAA) was performed by adding 100 µL of 1% L-FDAA. It is important to explain that in the total hydrolysis conditions used, the pyroglutamic acid residue is transformed into glutamic acid. The resulting L-FDAA derivatives of all amino acids (Glu, Pro, Leu, Thr) were dissolved in MeOH (500 μL) for subsequent analysis. Authentic standards of L-Glu, L-Pro, L-Leu, and L-Thr were treated with L-FDAA and D-FDAA using the same procedure described above. L-*allo*-Thr was also derivatized with Marfey’s reagent, to distinguish a threonine residue from an *allo*-Thr residue. By comparing the retention times recorded by high-resolution LC-MS analysis (Figure S13) of the obtained L-FDAA derivatives of Glu, Pro, Leu, and Thr with those of the authentic standards, it was possible to confirm the L-configuration for all amino acids, as well as the unambiguous presence of threonine.

Although the linear structure of **1** would imply high conformational freedom, the observed dipolar couplings between remote protons could suggest a structured conformation. Concerning the *cis*/*trans* conformation of the peptide bond between prolines and the preceding amino acids, the *trans* conformation was suggested based on the Cβ and Cγ shift difference (Δδ^βγ^) that is below the threshold of 8 ppm [32]. The Cγ chemical shift above 23 ppm and the absence of the NOE correlation between the proline Hα and the Hα of the preceding amino acid further circumstantiated this assignment.

The high-resolution ESI mass spectrum of compound **2** showed the [M + H]^+^ ion peak at *m*/*z* 534.2915, which defined its molecular formula as C_26_H_39_N_5_O_7,_ requiring 10 degrees of unsaturation (Figure S14). The MS/MS spectrum of **2** suggested a linear pentapeptide structure, with the *b_4_* fragment arising from the neutral loss of a terminal proline. The observed ion series generated after the fragmentation of **2** (Figure S15) as well as the mass difference between peptides **1** and **2**, clearly indicated compound **2** to have the same amino acid sequence as **1**, but lacking the terminal Thr. A full set of homonuclear and heteronuclear two-dimensional NMR spectra was recorded for compound **2** (Figures S16–S20), thus confirming our observations. Only five α-proton signals were detected in the ^1^H NMR spectrum, suggesting a pentapeptide. The ^1^H NMR spectrum in conjunction with 2D COSY, TOCSY, HSQC, and NMR data, showed great similarity to those of **1** (Table S2), suggestive of a similar linear peptide to compound **1** lacking Thr. The amino acid sequence analysis was conducted similarly to that for **1**, by the careful analysis of HMBC NMR spectra. Based on the HMBC NMR correlations, a linear pGlu-Pro^I^-Leu-Pro^II^-Pro^III^ pentapeptide was confirmed for compound **2**.

### 2.5. Structural Prediction of the Proline-Rich Peptides ***3***–***8*** from Microbacterium sp. V1

De novo sequencing by MS/MS was attempted, to identify the unknown peptides **3**–**8** (Figures S21–S26), as being produced in very tiny amounts for purification and subsequent 2D NMR-based structure elucidation. Compounds **3**, **4**, and **6**–**8** were present in the SPE fraction where peptides **1** and **2** were eluted, i.e., fraction B, while compound **5** was eluted mainly with 75% MeOH during the SPE purification. Compounds **3** and **4** are two isomeric linear proline-rich nonapeptides, showing the same amino acid composition but different sequences and retention times. The high-resolution ESI mass spectrum of compounds **3** and **4** showed the [M + H]^+^ and [M + 2H]^2+^ ions at *m*/*z* 1025.5657 and *m*/*z* 513.2861, respectively, thus accounting for the molecular formula C_50_H_76_N_10_O_13_. The fragmentation pattern observed in the MS^2^ spectrum of the [M + H]^+^ and [M + 2H]^2+^ ions of compound 3 (Figure S21), displayed *b_8_*-*b_2_* and *y_7_*-*y_2_* fragment ions and internal fragments deriving from a combination of *b*- and *y-* type cleavage, which allowed for the determination of the amino acid sequence as Val-Val-Pro-Pro-Phe-Leu/Ile-Gln-Pro-Glu. Similarly, the sequence of compound 4 was established as Val-Pro-X-X-Leu/Ile-Gln-Pro-Glu-Val (Figure S22). The molecular formula of **4** was satisfied with the presence of one proline and one phenylalanine residue, which were tentatively assigned to the third and fourth positions of the peptide sequence, respectively. 

The high-resolution ESI mass spectrum of compound **5** featured the [M + H]^+^ pseudomolecular ion at *m*/*z* 724.4020, corresponding to the molecular formula C_37_H_53_N_7_O_8_. The product ion spectrum of **5** showed *b*_6_-*b*_4_ and *y*_6_-*y*_3_ fragment ions which, together with internal cleavage fragments, were used to infer the sequence of the linear heptapeptide as Pro-Phe-Pro-Gly-Pro-Ile/Leu-Pro (Figure S23A). Acquisition of the HR-MS^3^ spectrum of the fragment ions y_4_-H_2_O and the internal fragment Pro-Gly-Pro-Ile/Leu at *m*/*z* 365.2179 arising from fragmentation of **5**, allowed us to confirm sequence prediction (Figure S23B). Structures for compounds **1**–**8** are shown in Table 2. 

Putative structures of peptides **6**–**8** were obtained by analogy with compounds **3** and **4**, based on the assumption that compounds **6**–**8** derive from proteolytic degradation of **3** and/or **4**. Identification of the *b*-, *y*-, and *a*-type ion series as well as internal cleavage ions in the MS/MS spectra of peptides **6**–**8**, allowed us to establish that *(a)* compound **6** has the same amino acid sequence as **3**, but lacking the N-terminal Val (Figure S24), *(b)* compound **7** (Figure S25), which is isomeric with **6**, has the same amino acid sequence as **4**, but lacking the N-terminal Val, *(c)* compound **8** has the same amino acid sequence as **3**, but lacking the two N-terminal Val residues (Figure S26).

A preliminary screening of the de novo sequenced genome of *Microbacterium* V1 was performed by using the AntiSmash tool, to search for NRPS or RiPP gene cluster candidates encoding the biosynthesis of the proline-rich peptides **1**–**8**. However, the bacterial genome did not contain any NRP/RiPP biosynthetic pathway and, therefore, it was hypothesized that compounds **1**–**8** could be the products of the bacterial enzymatic degradation of larger endogenous or exogenous proteins. Therefore, a Blastp analysis of the amino acid sequence of peptides **1**–**8** was performed against the UniprotKB/Swiss-Prot database and the *Microbacterium* V1 proteome. Notably, all the peptides **1**–**8** were found to correspond to different fragments of the bovine β-casein (P02666), as highlighted in Table 2. The bovine β-casein digest (i.e., tryptone) is the main nitrogen source in the LB medium used for the growth of *Microbacterium* V1. Therefore, it can be argued that the bacterium can secrete proteolytic enzymes to generate oligopeptides from the β-casein digest, which are then imported and probably subjected to multiple hydrolytic steps in the bacterial cells, where they are accumulated. LC-HRMS analysis of the LB medium without any bacteria inoculated, did not show any traces of PRPs **1**–**8**, further confirming these peptides are produced by *Microbacterium* V1. While very tiny amounts of some of the PRPs **1**–**8** could be detected also in bacterial cultures grown in peptone-based media (MB and SV2), containing β-casein-derived polypeptides among many other animal-derived proteins, none of the compounds were produced when the bacterium was grown in the ISP2 medium, lacking either tryptone or peptone. Taken together, these findings provide an additional clue to the origin of PRPs **1**–**8** from bacterial-mediated hydrolysis of β-casein fragments. 

Based upon the assumption that peptides **1** and **2** correspond to fragments 164–169 and 164–168 of the bovine β-casein (Table 2), the presence of the non-canonical pyroglutamic acid in **1** and **2** is likely to derive from the spontaneous intramolecular cyclization of the Gln164 residue, as a glutaminyl *cyclase* homologue could not be found in the *Microbacterium* V1 genome. In addition, the analogy of peptides **3**–**8** with fragments of the bovine β-casein allowed us to distinguish between the isomeric Ile and Leu residues and establish the sequence of **4** as Val-Pro-Pro-Phe-Leu-Gln-Pro-Glu-Val, with the Pro and Phe residues assigned, respectively, to the third and fourth position of the peptide sequence.

Interrogation of the BIOPEP database available at https://biochemia.uwm.edu.pl/en/biopep-uwm-2/ (accessed on 15 February 2023) [33,34] disclosed peptides **4** [14] and **6** [35] to be reported bioactive peptides from fermented β-casein, endowed with anti-inflammatory and dipeptidyl-peptidase 4 (DPP-4)-inhibitory activity, respectively. 

### 2.6. Assessment of Antioxidant Activity of Pure Metabolites

The fully characterized peptides **1** and **2** were subjected to a deep screening of bioactivities including antimicrobial, antibiofilm, and antiviral, and showed poor results. However, the observation of iron (II) adducts in the full MS spectra of **1** and **2** (Figure S27), suggested the potential capacity of these molecules to reduce iron. Ferrous iron is known to trigger lipid peroxidation by the Fenton reaction and generate peroxyl and alkoxyl radicals by degradation of lipid hydroperoxides [36,37]. Lipid oxidation products may cause cell membrane damage as well as detrimental oxidation of nutrients [38,39]. The FRAP assay is an efficient and sensitive assay to establish the antioxidant power of a molecule as its iron-reducing capacity [36,40] and has been widely applied in nutritional science and in the agri-food industries to measure the “total antioxidant content” of foods [41].

The FRAP assay was performed on both peptides **1** and **2**, by using ascorbic acid as the positive control. During this assay, absorbance is monitored at the typical λ = 593 nm in order to detect the formation of the reduced form of the dye, the iron complex tripyridyltriazine (TPTZ). Compounds **1** and **2** were shown to possess antioxidant activity in the form of iron-reducing capacity, confirming our MS observations. In particular, these peptides seemed to have slower kinetics compared to ascorbic acid, with FRAP values respectively increasing from 41 ± 1 and 80 ± 2 at 4 min to 51 ± 2 and 121 ± 4 at 16 min (Table S3). It is known that strong antioxidants such as ascorbic acid or Trolox reach their absorbance plateau in a few seconds, and their absolute maximum is commonly read after 4 min. However, different molecules might possess slower kinetics as in the case of peptides **1** and **2**.

## 3. Materials and Methods

### 3.1. General Experimental Procedures

^1^H NMR and 2D NMR experiments were performed at 700 and 600 MHz on a Bruker Avance Neo spectrometer (Bruker BioSpin Corporation, Billerica, MA, USA) using CD_3_OD as solvent. All chemical shifts were referenced to the residual solvent signal (δ_H_ 3.31, δ_C_ 49.0). Through-space ^1^H connectivities were evidenced using a ROESY experiment with a mixing time of 200 ms. The HSQC spectra were optimized for ^1^*J*_CH_ = 145 Hz and the HMBC experiments for ^2,3^*J*_CH_ = 8.0 Hz. High-resolution MS and LC-MS experiments were recorded on a Thermo LTQ Orbitrap XL mass spectrometer (Thermo Fisher Scientific Inc., Waltham, MA, USA) combined with a Thermo U3000 HPLC system equipped with a solvent reservoir, inline degasser, binary pump, and refrigerated autosampler. The purification was performed on an HPLC Jasco featuring a quaternary pump, equipped with a Jupiter C18 analytical column (5 μM, 250 mm × 4.6 mm i.d.) and the metabolites were revealed by using a photodiode array detector (PDA).

UV-Vis measurements at 593 nm were performed with the BioTek Synergy HTX Multimode Reader (Agilent Technologies, Santa Clara, CA, USA).

### 3.2. Media and Buffers

LB: tryptone 10 g/L, yeast extract 5 g/L, NaCl 10 g/L.

MB: Marine Broth (Condalab, Madrid, Spain).

ISP2: glucose 4 g/L, yeast extract 4 g/L, malt extract 10 g/L.

SV2: glucose 15 g/L, peptone 15 g/L, glycerol 15 g/L, CaCO_3_ 1 g/L.

All the media ingredients were dissolved in distilled water and autoclaved before use.

### 3.3. OSMAC-Based Cultivation and Extraction Procedures

*Microbacterium* sp. V1 was cultivated in eight different culture conditions following the OSMAC approach. A single CFU of V1 was inoculated in 3 mL of four different media (LB, MB, ISP2, and SV2) at 25 °C for 24 h. Then, each inoculum was used to inoculate 2 × 100 mL flasks (20 mL of culture) of the respective medium at 0.01 OD_600_/mL. One flask of each medium was then incubated at 15 °C, while the other was incubated at 30 °C. Agitation (180 rpm) and time (5 days) were kept constant for all the cultivations.

At the end of the fifth day, all the cultures were centrifuged at 4 °C, thus obtaining a supernatant and a bacterial pellet for each condition. The pellets were extracted overnight with 100 mL of MeOH each, then centrifuged, and the upper organic phases were dried to afford the corresponding intracellular extracts. Differently, the supernatants were extracted with 2 volumes of EtOAc, and the organic phase was dried to afford the corresponding extracellular extract.

### 3.4. LC-HRMS and LC-HRMS^2^ Analysis of the Crude Extracts

For LC-HRMS and LC-HRMS^2^ analysis, the OSMAC extracts and the SPE fractions were dissolved at 2 mg/mL of mass-grade MeOH. MS runs were performed on a Thermo LTQ Orbitrap XL high-resolution ESI mass spectrometer coupled to a Thermo U3000 HPLC system. Experiments were performed with a Kinetex 5 µm 100 mm × 2.10 mm C18 column (Phenomenex, Torrance, CA, USA), kept at 25 °C. An elution gradient of H_2_O and MeOH running with a flow rate of 200 μL/min was used. The gradient program was as follows: 10% MeOH for 1 min, 10−100% MeOH over 30 min, and 100% MeOH for 10 min. Mass spectra were acquired in positive ion detection mode, with resolution set to 60,000 in the range of *m*/*z* 150–2000. Optimized MS parameters: spray voltage 4.80 kV, capillary temperature 285 °C, sheath gas rate 32 units N_2_ (ca. 320 mL/min), auxiliary gas rate 15 units N_2_ (ca. 150 mL/min). MS/MS spectra were obtained in the data-dependent acquisition (DDA) mode, in which the four most intense ions in the full-scan mass spectrum were subjected to high-resolution tandem mass spectrometry (HRMS^2^) analysis. HRMS^2^ spectra of the selected ions were collected with collision-induced dissociation (CID) fragmentation, an isolation width of 3.00 Da, a normalized collision energy of 35 units, an activation Q of 0.250 units, and an activation time of 30 ms. Mass data were analyzed using the Thermo Xcalibur software version 2.2 (Thermo Fisher Scientific Inc., Waltham, MA, USA).

### 3.5. Large-Scale Cultivation and Extract Fractionation and Purification

As result of metabolomics analysis on the OSMAC extracts, the cultivation in LB at 15 °C was upscaled and 6 × 3 L flasks containing 500 mL of media each were inoculated at 0.01 OD_600_/mL V1 concentration and 180 rpm. The crude intracellular extract containing the compounds of interest, was obtained as previously described and subjected to SPE fractionation by employing a Chromabond C-18 column cartridge (Macherey Nagel, Duren, Germany). The sample was dissolved in the minimum amount of MeOH and uploaded on the column head, the elution was performed by using two column volumes of each eluent solution, here reported in the order of use: (A) 100% H_2_O, (B) 75% H_2_O—25% MeOH, (C) 50% H_2_O—50% MeOH, (D) 25% H_2_O—75% MeOH, (E) 100% MeOH. This procedure afforded Fraction A (170 mg), Fraction B (27 mg), Fraction C (23 mg), Fraction D (3.6 mg), and Fraction E (530 mg).

Fraction B, which was enriched in the compounds of interest, was further purified with a UPLC equipped with a Jupiter C18 analytical column. The mobile phase was operated at a constant flow rate of 1 mL/min and was composed of different percentages of Buffer A (H_2_O + 0.1% TFA) and Buffer B (MeOH + 0.1% TFA) following the gradient: initial 100% A—0% B; 0—30 min, 40% A—60% B; 30–31 min 0% A—100% B; 31–36 min 0% A—100% B.

This procedure afforded two pure compounds: compound **1** (2.9 mg) and compound **2** (0.6 mg).

### 3.6. Metabolomic and Cheminformatic Analysis

The thermo .raw data were uploaded on MZmine 2.53 [30], for data processing, producing two output files, a .mgf file containing MS data, and a .csv file with quantitative information.

The two files were employed as input on the GNPS platform [23] to build a molecular network through the feature-based molecular networking (FBMN) path [42]. The precursor ion mass and the MS/MS fragment tolerances were both set to 0.02 Da. The maximum number of nodes in one cluster was set to 100, minimum cosine score for nodes linking was 0.7 with a minimum of three matching peaks. Analogs were also searched, not exceeding the difference of 300 Da between the query and the hit; the analogs had to follow the same clustering rules as mentioned above. For network visualization, Cytoscape v3.8.0 was employed.

The SPE fractions FBMN job can be publicly accessed at: https://gnps.ucsd.edu/ProteoSAFe/status.jsp?task=2f6182d0de274c8d88e40db49d0a74ff.

FBMN output was further run with the MolNetEnhancer workflow [43], which makes use of the ClassyFire algorithm to perform an automated chemical classification of all the clusters present in the network if possible.

### 3.7. Advanced Marfey’s Analysis

An amount of 100 μg of compound **1** was subjected to total hydrolysis with 500 μL 6 N HCl/AcOH (1:1) at 120 °C for 1 h. After the removal of the residual HCl fumes under a direct N_2_ flux, the hydrolysate of **1** was dissolved in TEA/acetone (2:3, 100 μL). 100 μL of 1% 1-fluoro-2,4-dinitrophenyl-5-D-alaninamide (L-FDAA) in CH_3_CN/acetone (1:2) were added to the solution. The mixture was heated at 50 °C for 2 h and dried under N_2_ stream. It is necessary to point out that in the hydrolysis conditions used, the pyroglutamic acid residue is transformed into glutamic acid. The resulting L-FDAA derivatives of all amino acids (Glu, Pro, Thr, Leu) were dissolved in MeOH (500 μL) for subsequent analysis. Authentic standards of L-Glu, L-Pro, L-Leu, and L-Thr were treated with L-FDAA and D-FDAA by the same procedure as described above. To clarify the stereochemistry of the threonine residue, L-*allo*-Thr was also derivatized with Marfey’s reagent. The hydrolysate of compound **1** and standards were analyzed by RP-HPLC using a Kinetex C18 (Phenomenex) 150 × 4.6 mm, 2.6 μm column, kept at 25 °C and with an elution flow of 200 μL min^−1^ with 0.1% HCOOH in H_2_O and ACN. The retention times of Marfey’s derivatives of compound **1** were compared with those of the standard derivatives by HPLC-ESI-HRMS. The gradient program was as follows: prerun of 35 min at 5% ACN 3 min, 5% → 50% ACN over 30 min, 50% ACN 1 min, 50% ACN → −90% 6 min. Mass spectra were acquired in positive ion detection mode, and raw data were analyzed using the Xcalibur suite of programs.

### 3.8. FRAP Assay of Pure Metabolites 

The antioxidant capacity of the pure metabolites was assessed through the FRAP assay. This sensitive assay developed by Benzie and Strain [40] measures the capacity of a sample of reducing Fe^3+^ to Fe^2+^. In brief, the assay relies on the color change of the dye tripyridyltriazine iron complexes, Fe^3+^-TPTZ (colorless) to Fe^2+^-TPTZ (intense blue), which occurs when a compound capable of carrying out iron reduction is present. In brief, three solutions were prepared: (1) an acetate buffer 300 Mm with pH 3.6, (2) a 10 mM TPTZ solution dissolved in 40 mM HCl, and (3) a 20 mM FeC1_3_·6H_2_O solution. The working solution was prepared immediately prior to its utilization by mixing the three solutions in the ratios 10:1:1. The test was performed in a 96-well plate as previously described, by monitoring the reaction at 593 nm while keeping temperature constant at 37 °C [44]. Freshly prepared ascorbic acid was employed to calibrate the method (100, 250, 500, and 1000 μM). The samples (5 mM), and the positive control (ascorbic acid 100 μM) were dissolved in water, thus water was employed as the negative control. All measurements were performed in triplicate. The FRAP values were calculated from the ascorbic acid equivalents values using the following equation [44]:

FRAP value = [(A_1_ − A_0_)/(A_c_ − A_0_)] × 2, where A_c_ is the absorbance of the positive control, A_1_ is the absorbance of the sample, and A_0_ is the absorbance of the blank. Results were acquired after 30 s and then once every minute for 16 min for samples, ascorbic acid, and negative control.

## 4. Conclusions

Herein we present the investigation of the biotechnological potential of a marine strain belonging to the genus *Microbacterium* associated with the marine sponge *Petrosia ficiformis*. The OSMAC approach was employed as a strategy to expand *Microbacterium* sp. V1 metabolome, while a combination of untargeted metabolomics and cheminformatics allowed a straightforward dereplication and prioritization of the produced metabolites. Experimental evidence showed an improved production of a pool of PRPs (**1**–**8**), majorly accumulated inside the cell, in the LB-15 °C culture condition. Therefore, after culture upscaling, two unprecedented linear PRPs containing an N-terminal pyroglutamic acid residue could be isolated and stereo-structurally elucidated through a complete set of NMR experiments. Both compounds displayed good antioxidant activity in the FRAP assay, with compound **2** being approximately twice as effective than **1.**

Interestingly, all the PRPs **1**–**8** were found to correspond to different fragments of the bovine β-casein, thereby suggesting the ability of *Microbacterium* sp. V1 to release hidden bioactive oligopeptides by using peptidases for the catabolism of proline-containing proteins. To date, such proteolytic systems, involving both extra- and intracellular proteases, have been characterized in detail only in lactic acid bacteria [45]. However, annotation of the proteome of *Microbacterium* sp. V1 by the tool Hotpep-protease [46], led us to detect more than 100 putative proteases, with 80% being represented by serine- and metallopeptidases (Figure S28). Unlocking the function and substrate selectivity of the proteases releasing the PRPs from *Microbacterium* sp. V1 will be the aim of our future work, which could unveil an additional way to generate small peptides in sponge microbial communities, besides the canonical RiPP and NRPS pathways.

Overall, our results highlight the promising biotechnological potential of *Microbacterium* sp. V1 as a factory for the production of bioactive PRPs.

## Figures and Tables

**Table 1:** Full NMR data of compound (**1**) (^1^H 700 MHz, ^13^C 175 MHz, CD_3_OD).

AA	Pos.	δ_C_, Type		δ_H_, Mult (*J* in Hz)	ROESY	HMBC ^a^
pGlu	NH					
	1	172.95, C				
	2	56.25, CH		4.56, dd (4.2, 8.9)	Pro^I^-5a, Pro^I^-5b	pGlu-1, pGlu-5
	3	25.51, CH_2_	a	2.50, m		pGlu-1, pGlu-5
			b	2.19, m		pGlu-1, pGlu-5
	4	30.31, CH_2_	a	2.40, m		pGlu-5
			b	2.31, m		pGlu-5
	5	181.55, C				
Pro^I^	1	174.23, C				
	2	61.60, CH		4.47, dd (4.3, 8.5)		pGlu-1, Pro^I^-1
	3	30.33, CH_2_	a	2.18, m		Pro^I^-1
			b	1.97, m		Pro^I^-1
	4	25.94, CH_2_	a	2.05, m		
			b	1.98, m		
	5	48.06, CH_2_	a	3.68, m	pGlu-2	pGlu-1
			b	3.61, m	pGlu-2	pGlu-1
Leu	NH					
	1	172.91, C				
	2	51.09, CH		4.62, t (7.2)	Pro^II^-5a, Pro^II^-5b	Leu-1, Pro^I^-1, Pro^I^-4
	3	41.04, CH_2_		1.56, t (7.2)		Leu-1
	4	25.76, CH		1.81, nonet (6.6)		
	5	21.86, CH_3_		0.97, d (6.6)		
Pro^II^	1	172.75, C				
	2	59.62, CH		4.69, dd (4.5, 8.3)	Pro^III^-2,Pro^III^-5a, Pro^III^-5b	Leu-1, Pro^II^-1
	3	29.41, CH_2_	a	2.27, m		Pro^II^-1
			b	2.04, m		Pro^II^-1
	4	25.87, CH_2_	a	2.13, m		
			b	2.01, m		
	5	48.56, CH_2_	a	3.84, m	Leu-2	Leu-1
			b	3.65, m	Leu-2	Leu-1
Pro^III^	1	174.66, C				
	2	61.47, CH		4.55, m	Pro^II^-2	Pro^II^-1, Pro^III^-1
	3	30.29, CH_2_	a	2.21, m		Pro^III^-1
			b	2.08, m		Pro^III^-1
	4	25.90, CH_2_	a	2.07, m		
			b	2.04, m		
	5	48.50, CH_2_	a	3.80, m	Pro^II^-2	Pro^II^-1
			b	3.67, m	Pro^II^-2	Pro^II^-1
Thr	NH					
	1	173.48, C				
	2	59.13, CH		4.38, d (2.9)		Pro^III^-1, Thr-1
	3	68.66, CH		4.29, dd (2.9, 6.4)		Thr-1
	4	20.51, CH_3_		1.20, d (6.4)		Thr-1

^a^ diagnostic correlations.

**Table 2:** Structures of peptides **1**–**8** from *Microbacterium* sp. V1.

Peptide	Sequence	*R*_t_ (min)	*m*/*z*	[M + H]^+^	β-Casein Fragment
1	pGlu-Pro-Leu-Pro-Pro-Thr	12.7	635.3392	C_30_H_47_N_6_O_9_	164–169
2	pGlu-Pro-Leu-Pro-Pro	12.0	534.2915	C_26_H_40_N_5_O_7_	164–168
3	Val-Val-Pro-Pro-Phe-Leu-Gln-Pro-Glu	17.3	1025.5657	C_50_H_77_N_10_O_13_	98–106
4	Val-Pro-Pro-Phe-Leu-Gln-Pro-Glu-Val	18.4	1025.5657	C_50_H_77_N_10_O_13_	99–107
5	Pro-Phe-Pro-Gly-Pro-Ile-Pro	17.4	724.4020	C_37_H_54_N_7_O_8_	76–82
6	Val-Pro-Pro-Phe-Leu-Gln-Pro-Glu	16.0	926.4953	C_45_H_68_N_9_O_12_	99–106
7	Pro-Pro-Phe-Leu-Gln-Pro-Glu-Val	17.0	926.4953	C_45_H_68_N_9_O_12_	100–107
8	Pro-Pro-Phe-Leu-Gln-Pro-Glu	14.2	827.4273	C_40_H_59_N_8_O_11_	100–106

## Data Availability

Data available in a publicly accessible repository that does not issue DOIs. These data can be found here: https://gnps.ucsd.edu/ProteoSAFe/status.jsp?task=2f6182d0de274c8d88e40db49d0a74ff.

## Supplementary Materials

The following data can be downloaded at https://www.mdpi.com/article/10.3390/md21040256/s1. Table S1: Mzmine parameters adopted for MS data processing; Figures S1–S12: MS, MS2, 1D and 2D NMR spectra of compound **1**; Figure S13: Advanced Marfey’s analysis of compound **1**; Table S2, Figures S14–S20: MS, MS2, 1D and 2D NMR spectra of compound **2**; Figure S21: HR-MS/MS spectrum of the [M + H]^+^ and [M + 2H]^2+^ ions of compound **3**; Figure S22: HR-MS/MS spectrum of the [M + H]^+^ and [M + 2H]^2+^ ions of compound **4.**; Figure S23: HR-MS/MS spectrum of the [M + H]^+^ pseudomolecular ion of compound **5.**; Figure S24: HR-MS/MS spectrum of the [M + H]^+^ and [M + 2H]^2+^ ions of compound **6**; Figure S25: HR-MS/MS spectrum of the [M + H]^+^ and [M + 2H]^2+^ ions of compound **7**; Figure S26: HR-MS/MS spectrum of the [M + H]^+^ and [M + 2H]^2+^ ions of compound **8**; Figure S27: HRMS spectra showing iron adducts of compounds **1** and **2**; Table S3: Ferric reducing antioxidant power assay results of compounds **1** and **2**; Figure S28: Peptidases from *Microbacterium* sp. V1 annotated by using the bioinformatic tool Hotpep-protease.
