# Supplementary material for: Enhanced Molecular Networking Shows Microbacterium sp. V1 as a Factory of Antioxidant Proline-Rich Peptides
Source: Mar Drugs. 2023 Apr 21;21(4):256. doi: 10.3390/md21040256 (PMC10146280; doi:10.3390/md21040256)
Supplement: Supplementary file 1 [file marinedrugs-21-00256-s001.zip › marinedrugs-2331100-supplementary.pdf]

marine drugs

Supplementary information

Enhanced Molecular Networking Shows *Microbacterium* sp. V1 as a Factory of Antioxidant Proline-Rich Peptides

Giovanni Andrea Vitale [1], Silvia Scarpato [2,3], Alfonso Mangoni [2], Maria Valeria D'Auria [2], Gerardo Della Sala [1]* and Donatella de Pascale [1]*

1 Department of Eco-Sustainable Marine Biotechnology, Stazione Zoologica Anton Dohrn, Via A.F. Acton, Molosiglio, 80133 Naples, Italy; giovanniandrea.vitale@szn.it (G.A.V);
2 Department of Pharmacy, University of Naples "Federico II", 80131 Naples, Italy; silvia.scarpato@unina.it (S.S.); alfonso.mangoni@unina.it (A.M.); madauria@unina.it (M.V.D.)
3 GEOMAR Centre for Marine Biotechnology (GEOMAR-Biotech), Research Unit Marine Natural Products Chemistry, GEOMAR Helmholtz Centre for Ocean Research Kiel, Am Kiel-Kanal 44, 24106 Kiel, Germany

* Correspondence: gerardo.dellasala@szn.it (G.D.S.); donatella.depascale@szn.it (D.d.P.)

Table S1. Mzmine parameters adopted for MS data processing.

Figure S1. Positive ion mode HR-ESI mass spectrum of compound **1**.

Figure S2. HR-MS/MS spectrum of the $[M+H]^+$ pseudomolecular ion of compound **1**.

Figure S3. ^{1}H-NMR spectrum of compound **1**, (700 MHz, CD_3OD).

Figure S4. ^{13}C-NMR spectrum of compound **1**, (175 MHz, CD_3OD).

Figure S5. COSY spectrum of compound **1**, (700 MHz, CD_3OD).

Figure S6. TOCSY spectrum of compound **1**, (700 MHz, CD_3OD).

Figure S7. ROESY spectrum of compound **1,** (700 MHz, CD_3OD).

Figure S8. HSQC spectrum of compound **1,** (700 MHz, CD_3OD).

Figure S9. Band-selective HMBC spectrum of compound **1,** (700 MHz, CD_3OD).

Figure S10. HMBC spectrum of compound **1,** (700 MHz, CD_3OD).

Figure S11. Band-selective HMBC spectrum of compound **1,** (700 MHz, CD_3OD).

Figure S12. Band-selective HMBC spectrum of compound **1,** (700 MHz, CD_3OD).

Figure S13. Advanced Marfey's analysis of compound **1**.

Table S2. Full NMR data of compound **2,** (^{1}H 700 MHz, ^{13}C 175 MHz, CD_3OD).

Figure S14. Positive ion mode HR-ESI mass spectrum of compound **2.**

Figure S15 HR-MS/MS spectrum of the $[M+H]^+$ pseudomolecular ion of compound **2**.

Figure S16. ^{1}H-NMR spectrum of compound **2,** (600 MHz, CD_3OD).

Figure S17. COSY spectrum of compound **2,** (700 MHz, CD_3OD).

Figure S18. TOCSY spectrum of compound **2,** (700 MHz, CD_3OD).

Figure S19. HSQC spectrum of compound **2,** (600 MHz, CD_3OD).

Figure S20. HMBC spectrum of compound **2,** (600 MHz, CD_3OD).

Figure S21. HR-MS/MS spectrum of the $[M+H]^+$ and $[M+2H]^{2+}$ ions of compound **3.**

Figure S22. HR-MS/MS spectrum of the $[M+H]^+$ and $[M+2H]^{2+}$ ions of compound **4.**

Figure S23. (A) HR-MS/MS spectrum of the $[M+H]^+$ pseudomolecular ion of compound **5.**

Figure S24. HR-MS/MS spectrum of the $[M+H]^+$ and $[M+2H]^{2+}$ ions of compound **6.**

Figure S25. HR-MS/MS spectrum of the $[M+H]^+$ and $[M+2H]^{2+}$ ions of compound **7.**

Figure S26. HR-MS/MS spectrum of the $[M+H]^+$ and $[M+2H]^{2+}$ ions of compound **8.**

Figure S27. HRMS spectra showing iron adducts of compounds **1** (A) and **2** (B).

Table S3. Ferric reducing antioxidant power assay results of compounds **1** and **2**.

Figure S28. Peptidases from *Microbacterium sp.* V1 annotated by using the bioinformatic tool Hotpep-protease.

Table S1. Mzmine parameters adopted for MS data processing.

**Mass Detection**	
MS1 Detection	5·10^4
MS2 Detection	1·10^2
**ADAP Chromatogram builder**	
Min group size in n° of scans	4
Group intensity threshold	5·10^4
Min highest intensity	5·10^4
m/z tolerance (ppm)	20
**Chromatogram deconvolution (Baseline cut-off)**	
Min peak height	5·10^4
Peak duration range (min)	0-3
Baseline level	5·10^4
m/z range for MS2 scan pairing (Da)	0.002
RT range for MS2 scan pairing (min)	0.3

**Join aligner**	
m/z tolerance (ppm)	15
Weight for mass	50
RT tolerance (min)	0.3
Weight for RT	50
**Peak filter**	
Keep only features with MSMS scan	V
**Gap Filling-Peak finder**	
Intensity tolerance	10%
m/z tolerance (ppm)	20
RT tolerance (min)	0.5

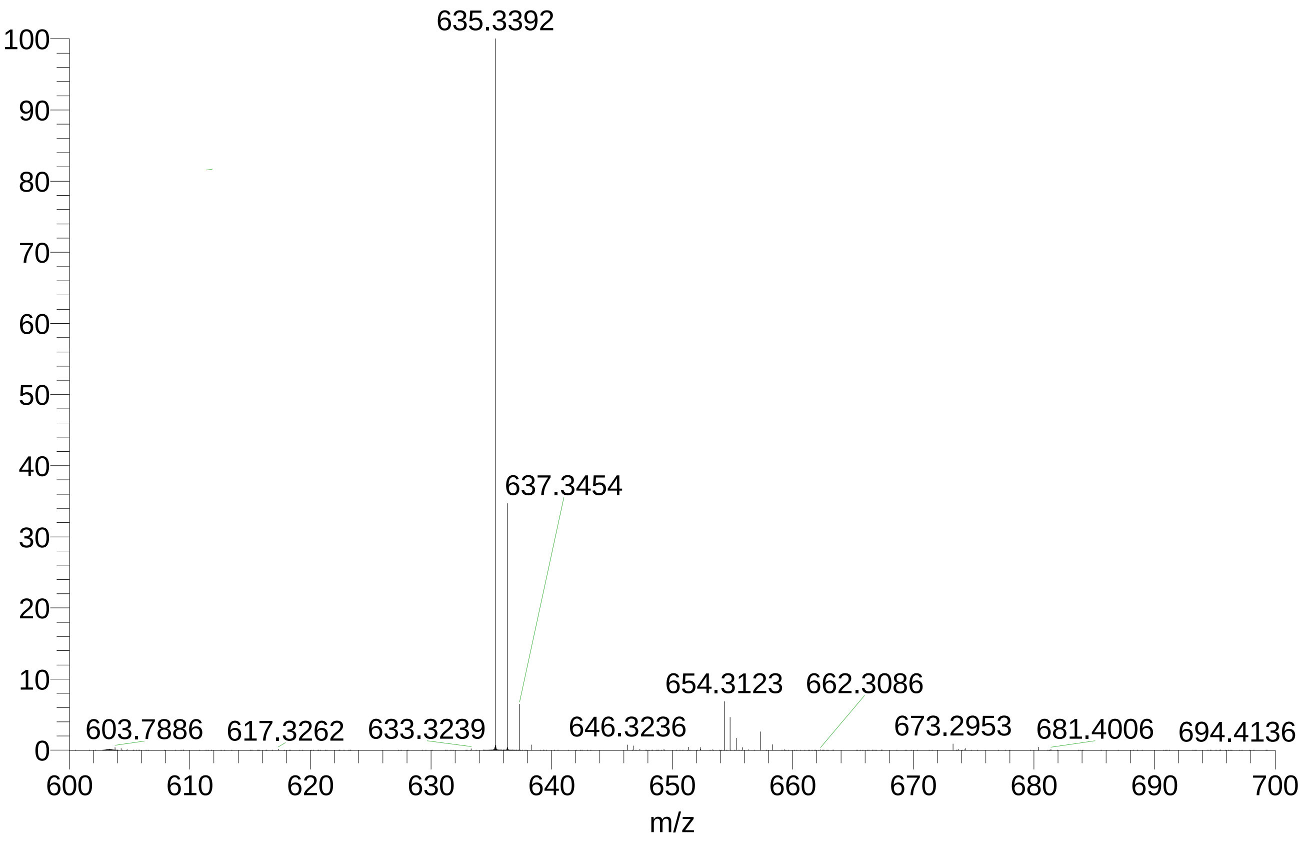

Figure S1. Positive ion mode HR-ESI mass spectrum of compound **1**.

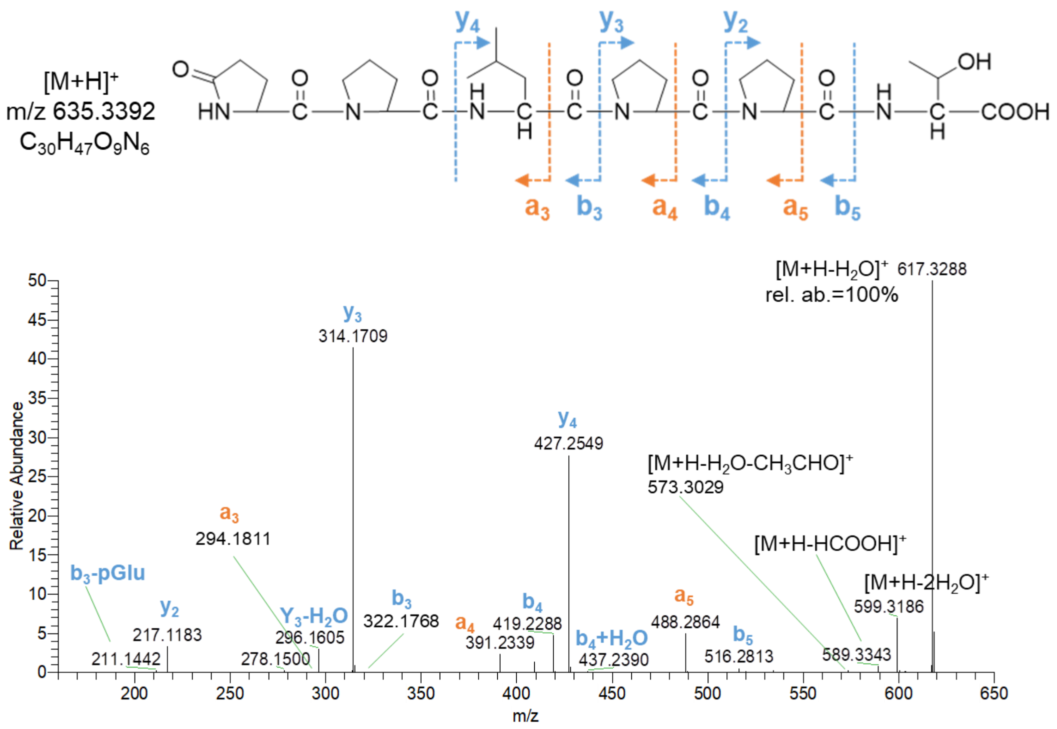

Figure S2. HR-MS/MS spectrum of the $[M+H]^+$ pseudomolecular ion of compound **1**.

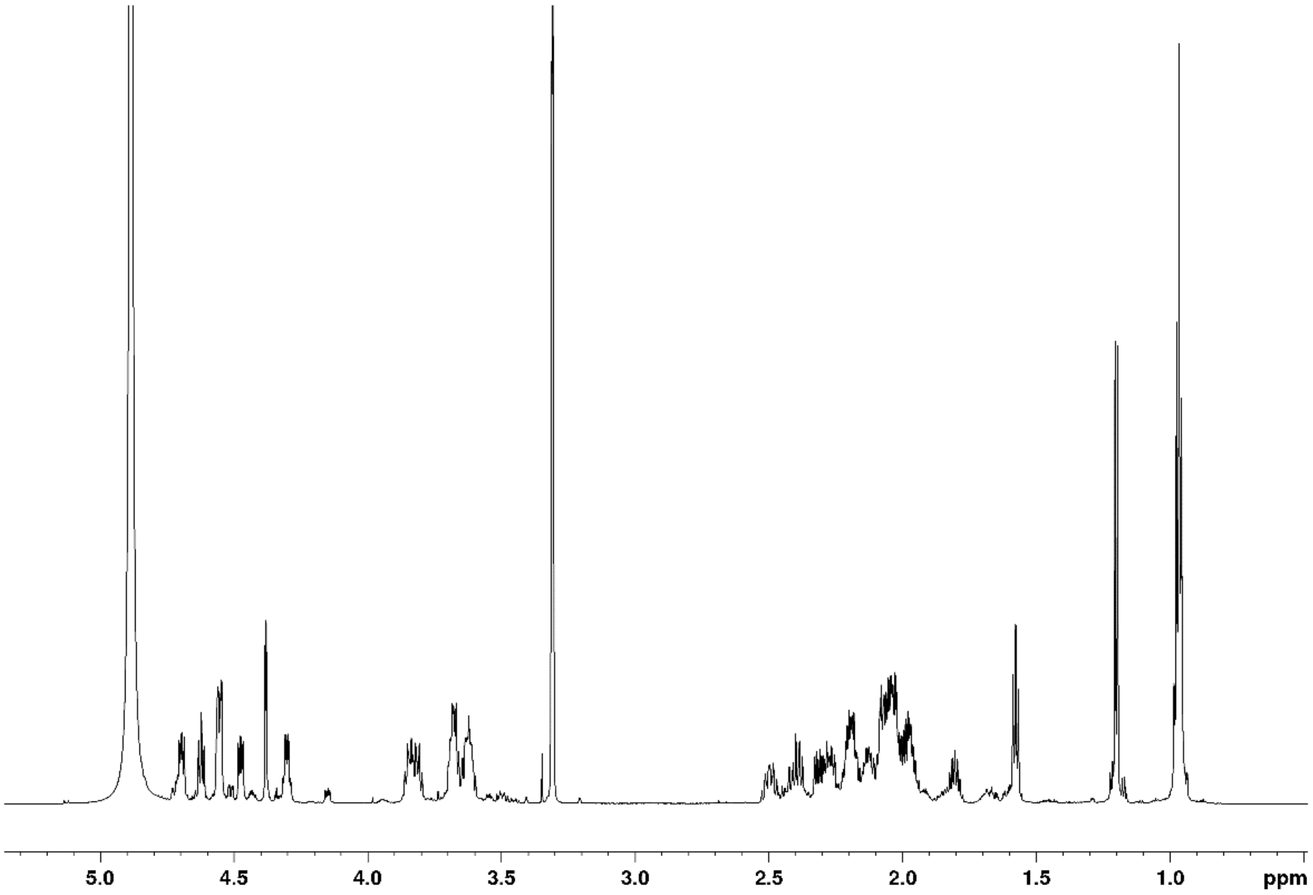

Figure S3. ^{1}H-NMR spectrum of compound **1** (700 MHz, CD_3OD).

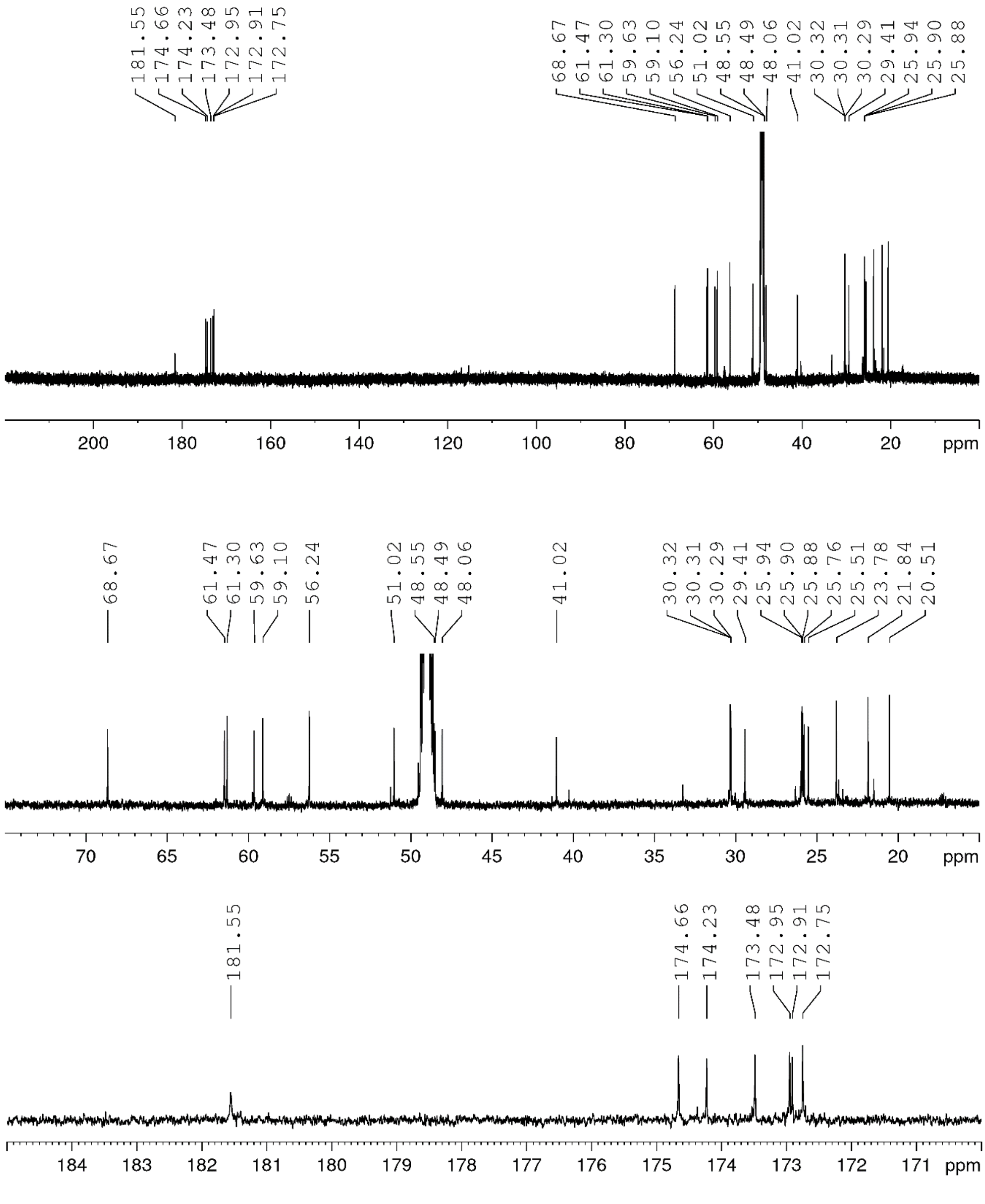

Figure S4. ^{13}C-NMR spectrum of compound **1** (175 MHz, CD_3OD).

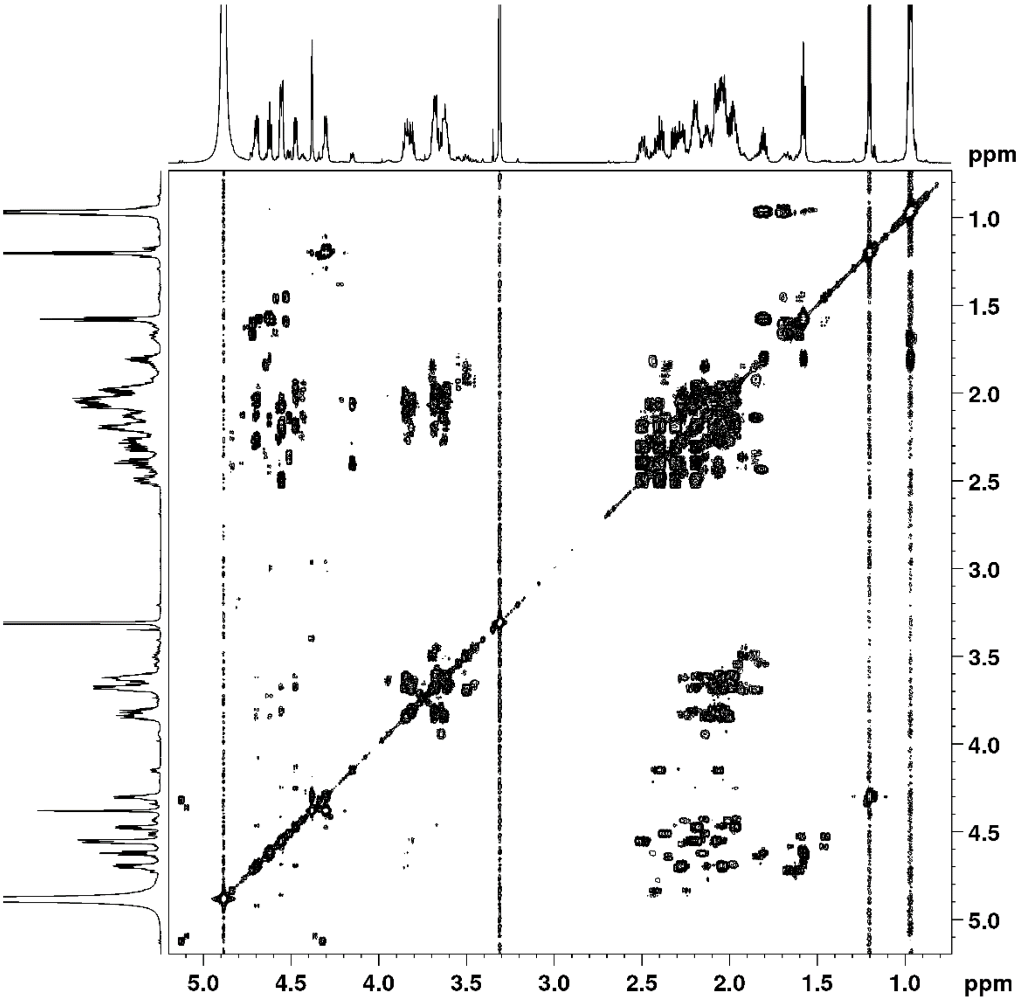

Figure S5. COSY spectrum of compound **1** (700 MHz, CD_3OD).

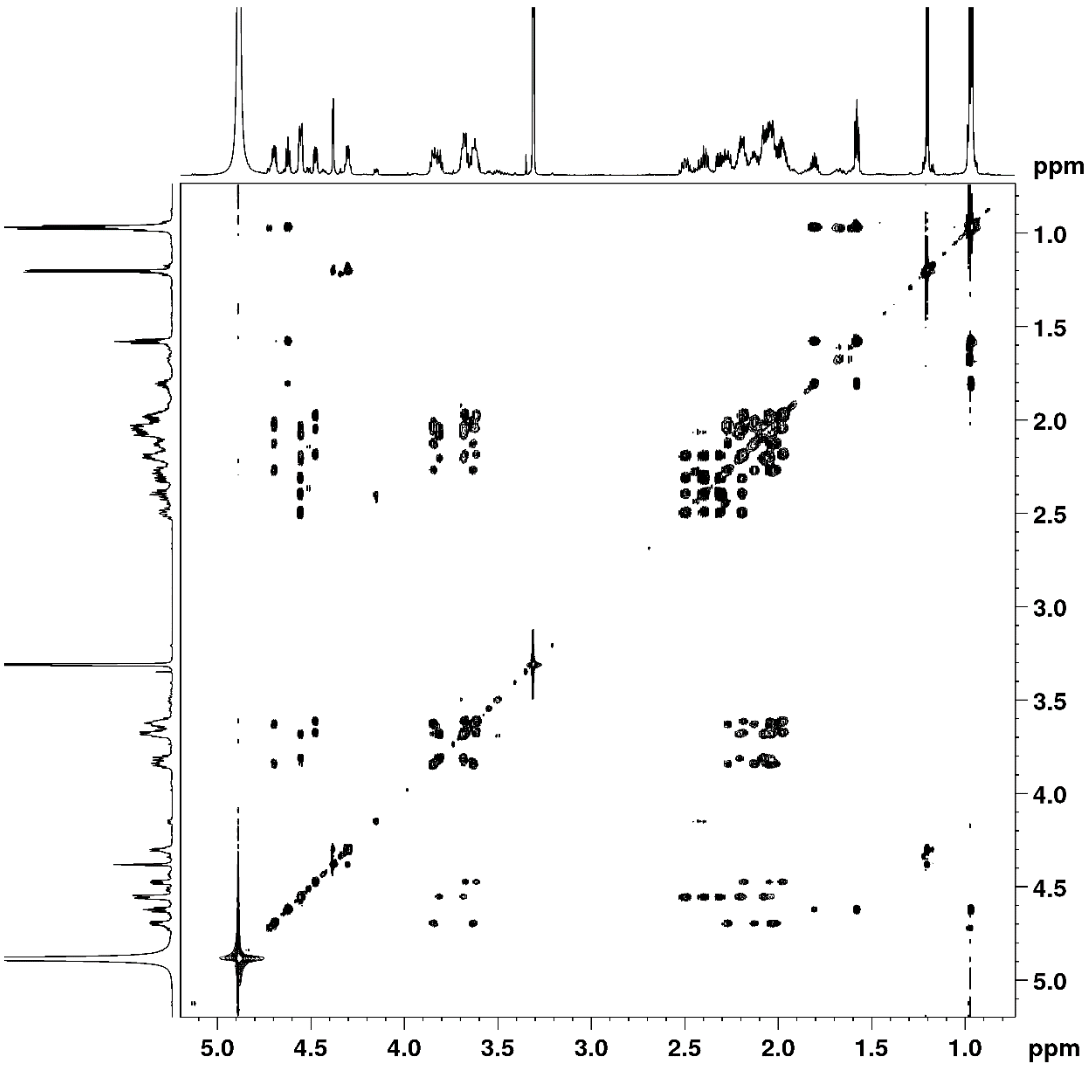

Figure S6. TOCSY spectrum of compound **1** (700 MHz, CD_3OD).

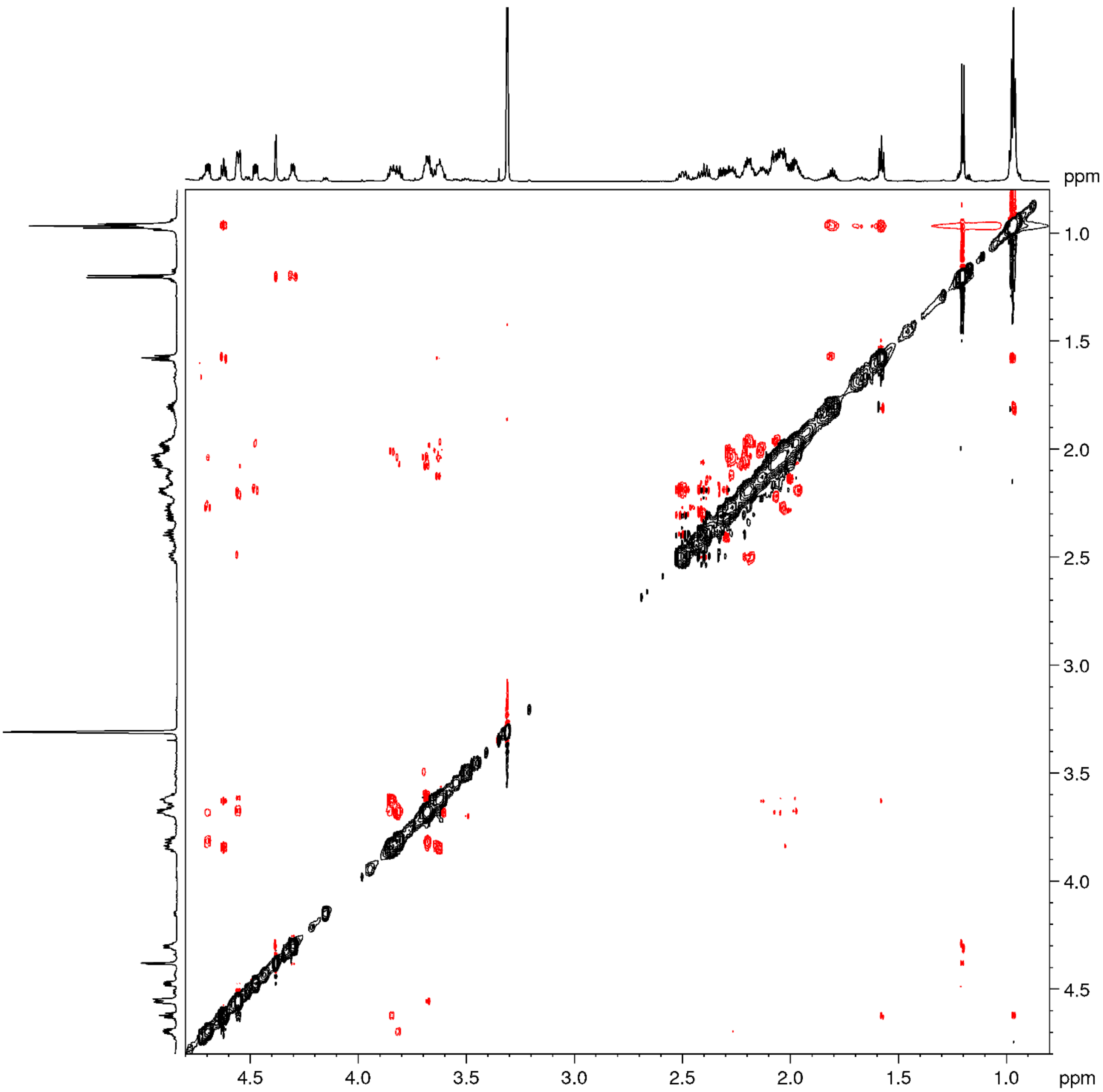

Figure S7. ROESY spectrum of compound **1** (**1**) (700 MHz, CD_3OD).

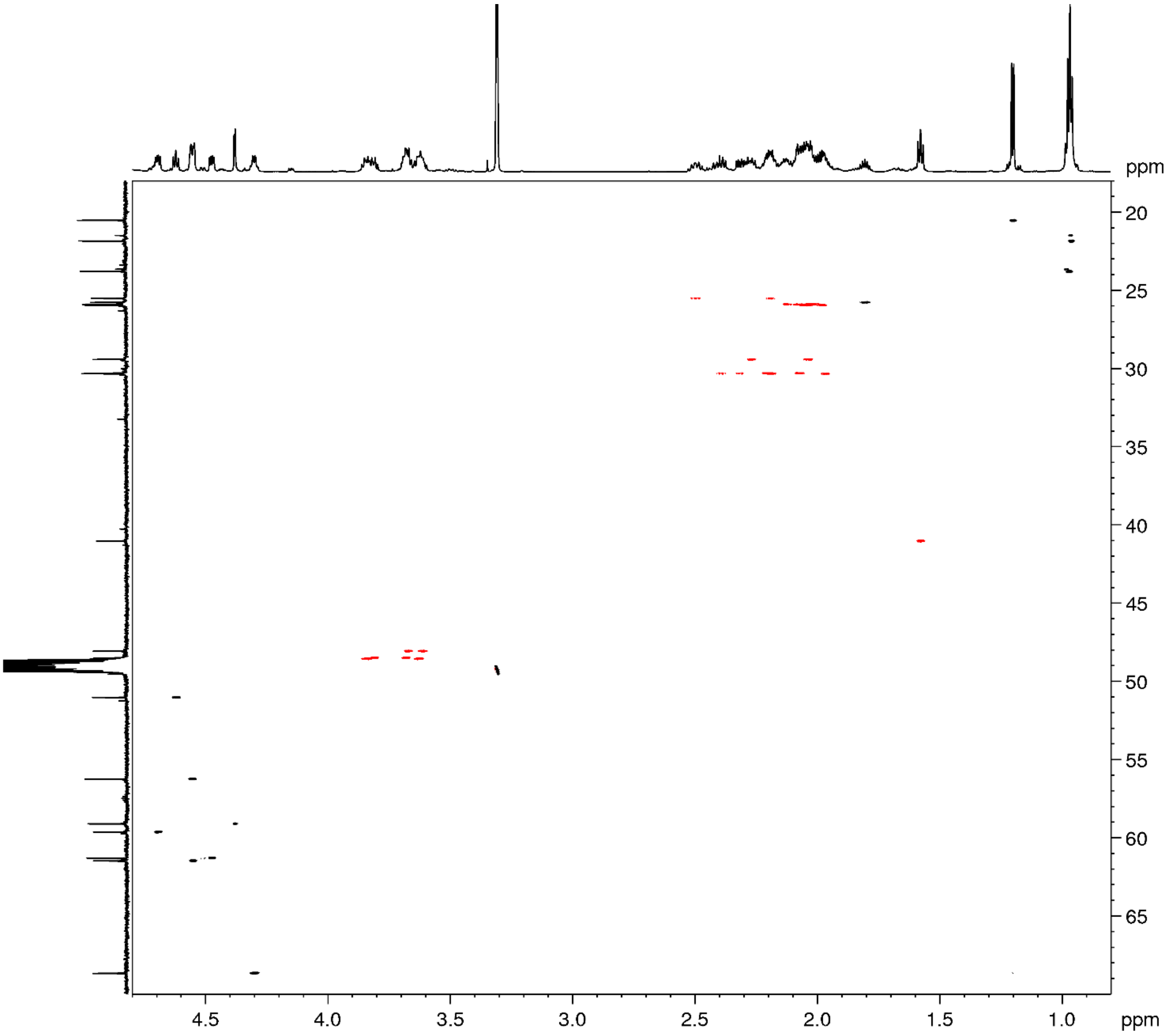

Figure S8. HSQC spectrum of compound **1** (700 MHz, CD_3OD).

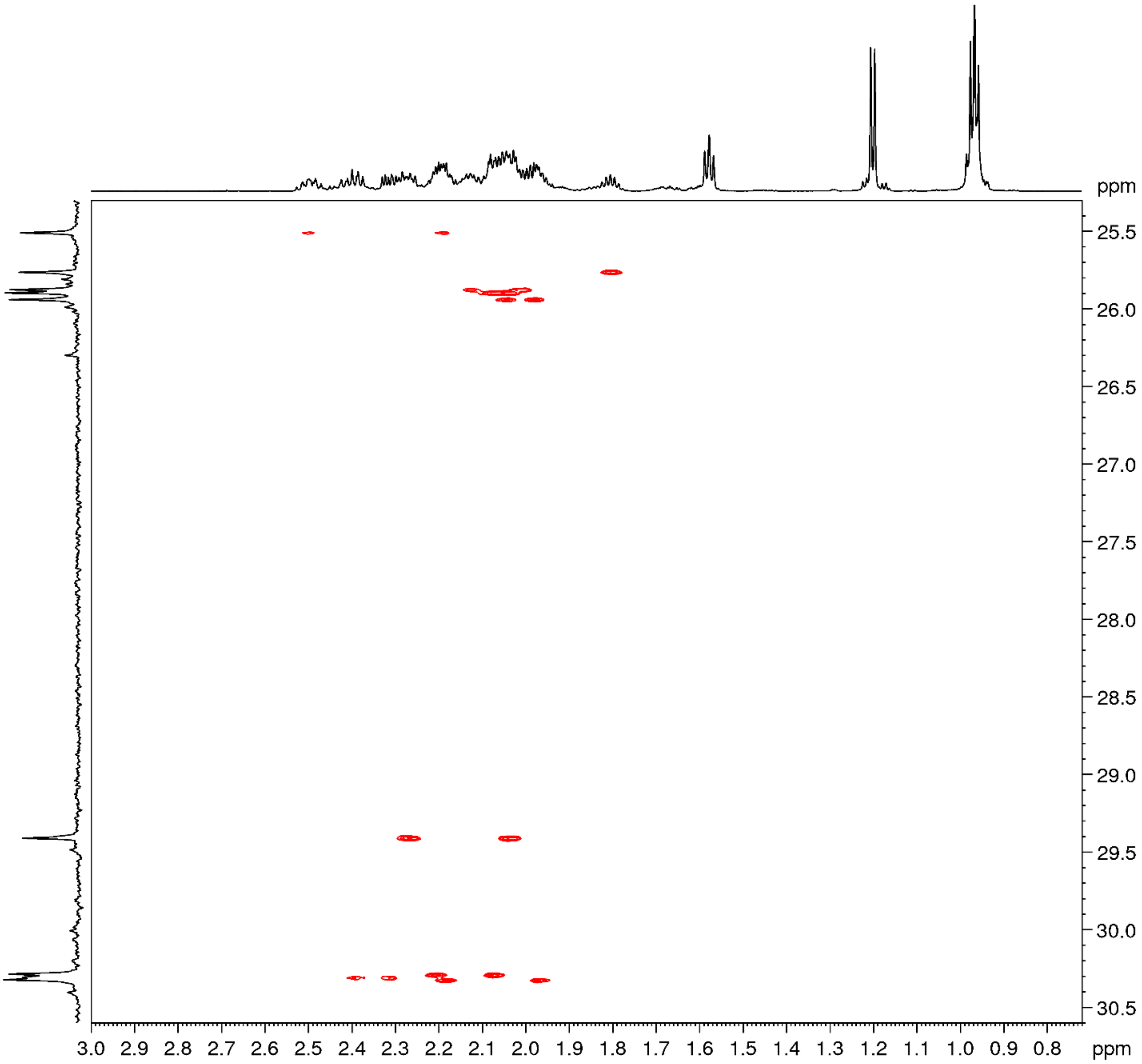

Figure S9. Band-selective HMBC spectrum of compound **1** (700 MHz, CD_3OD).

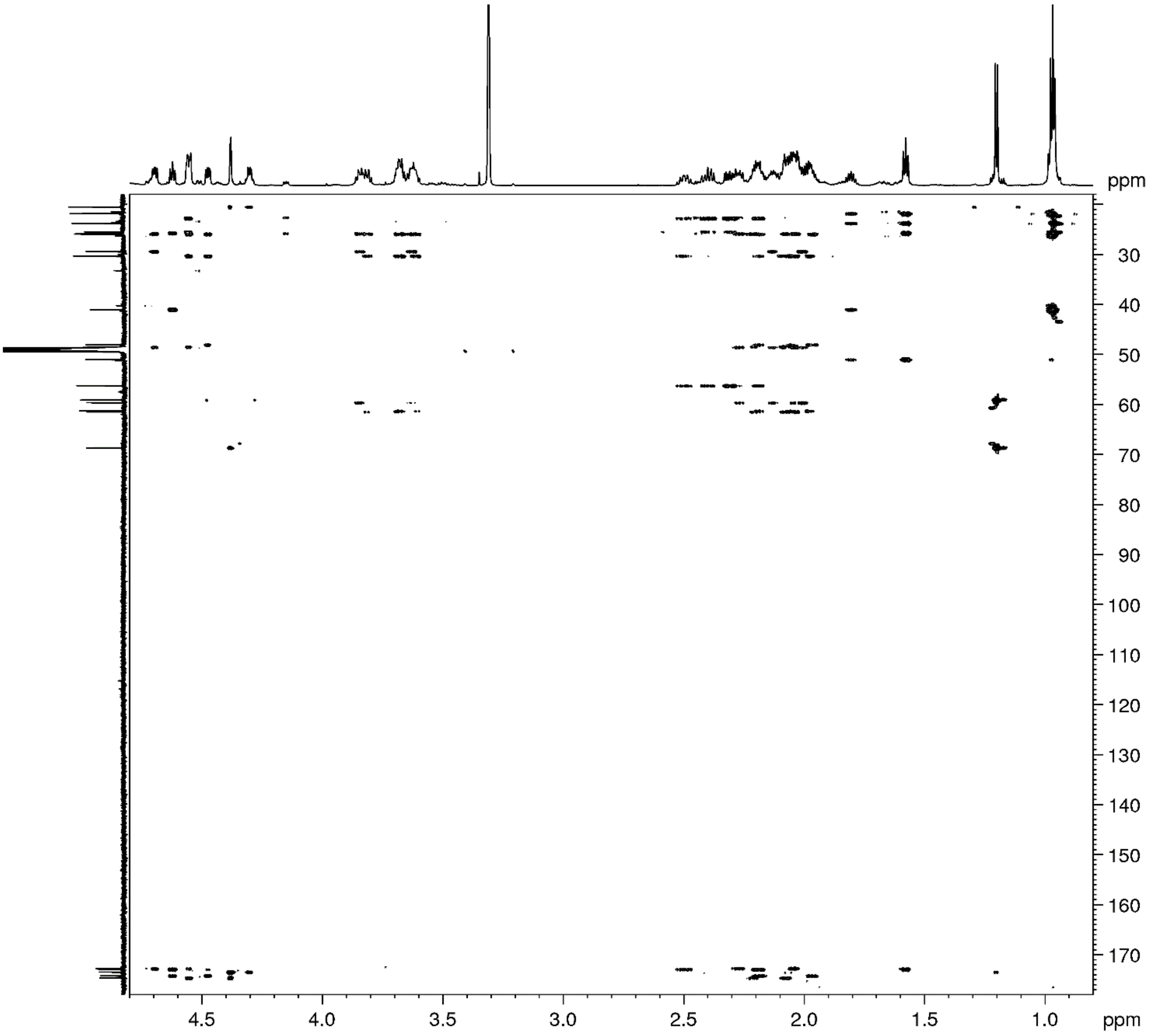

Figure S10. HMBC spectrum of compound **1** (700 MHz, CD_3OD).

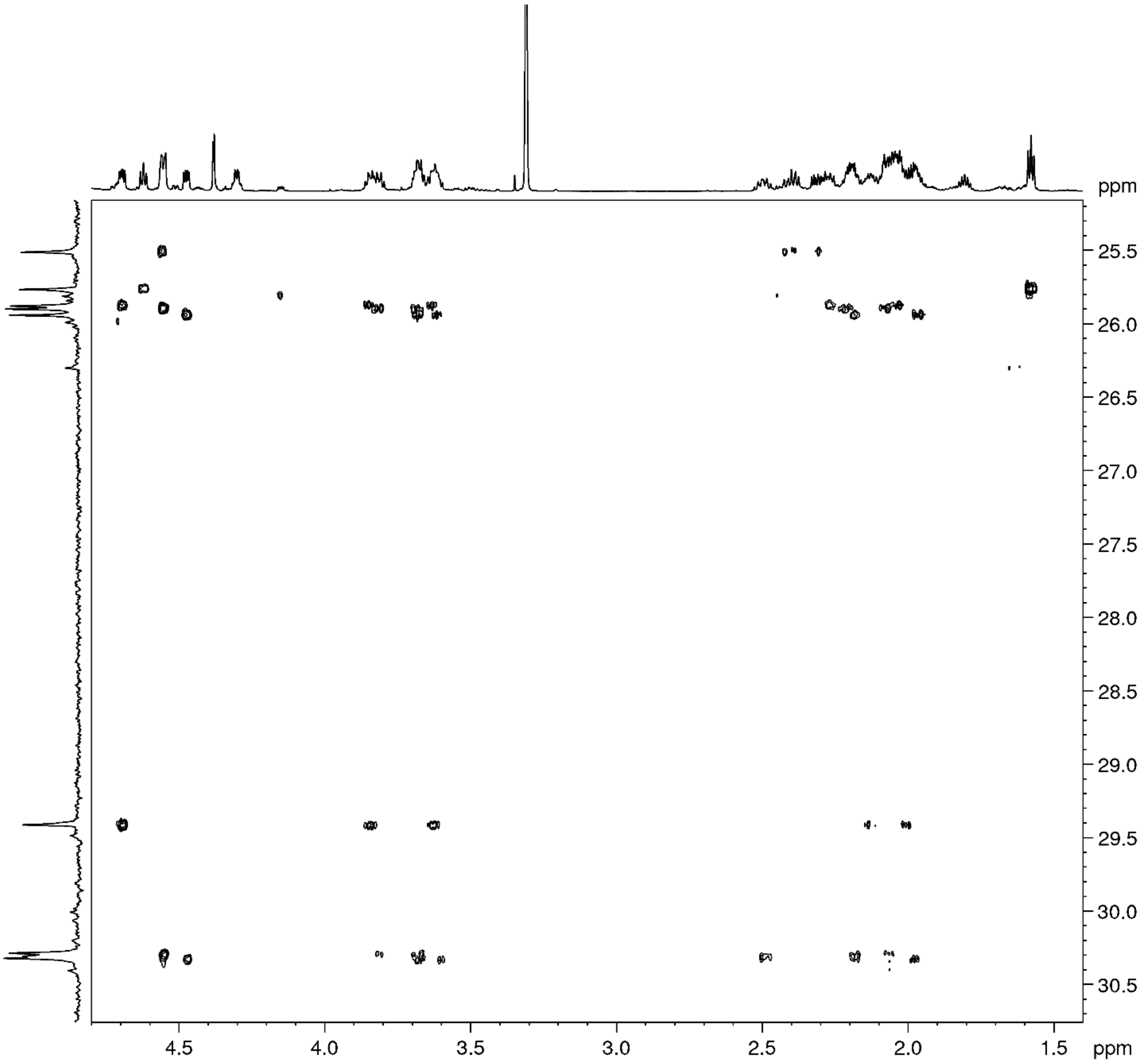

Figure S11. Band-selective HMBC spectrum of compound **1** (700 MHz, CD_3OD).

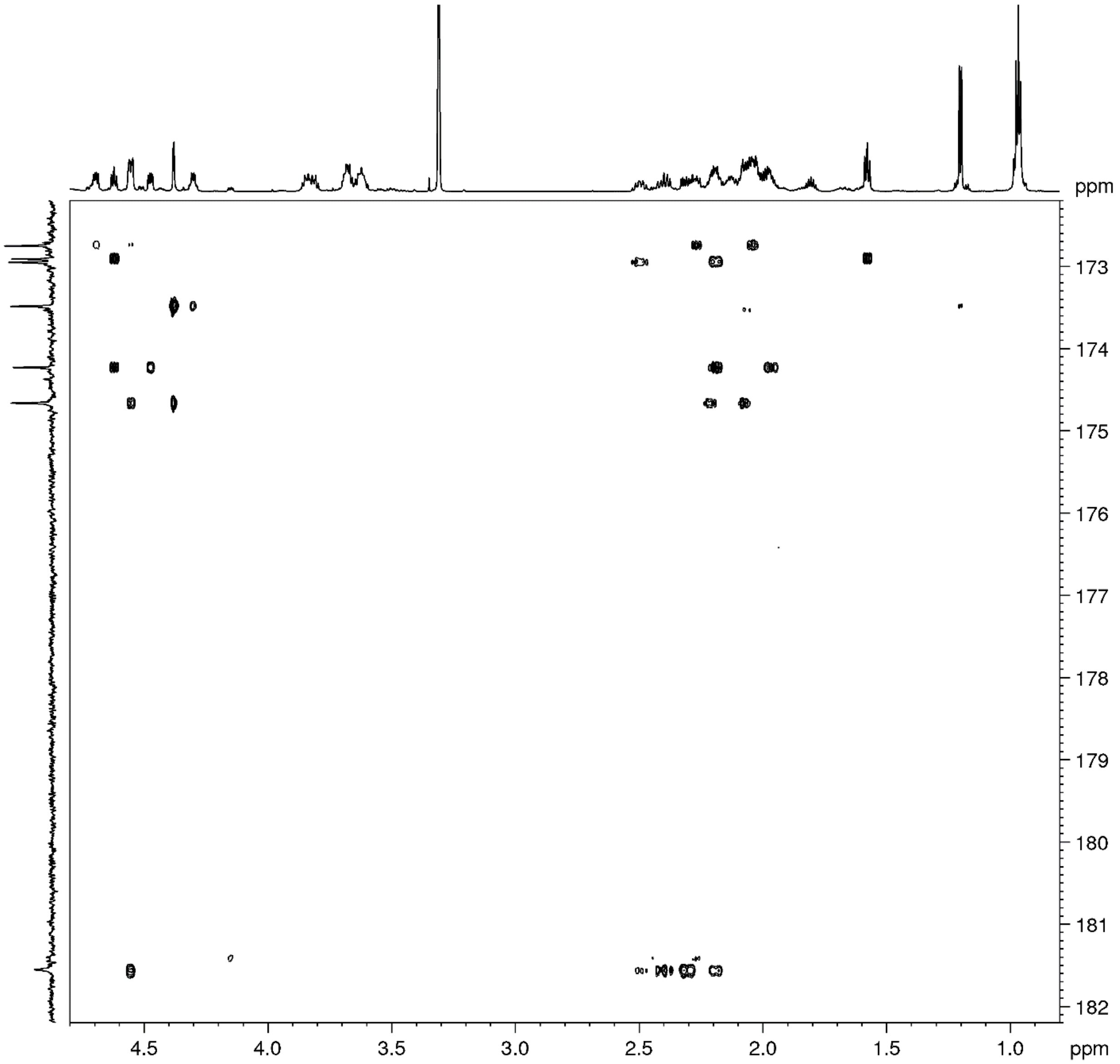

Figure S12. Band-selective HMBC spectrum of compound **1** (700 MHz, CD_3OD).

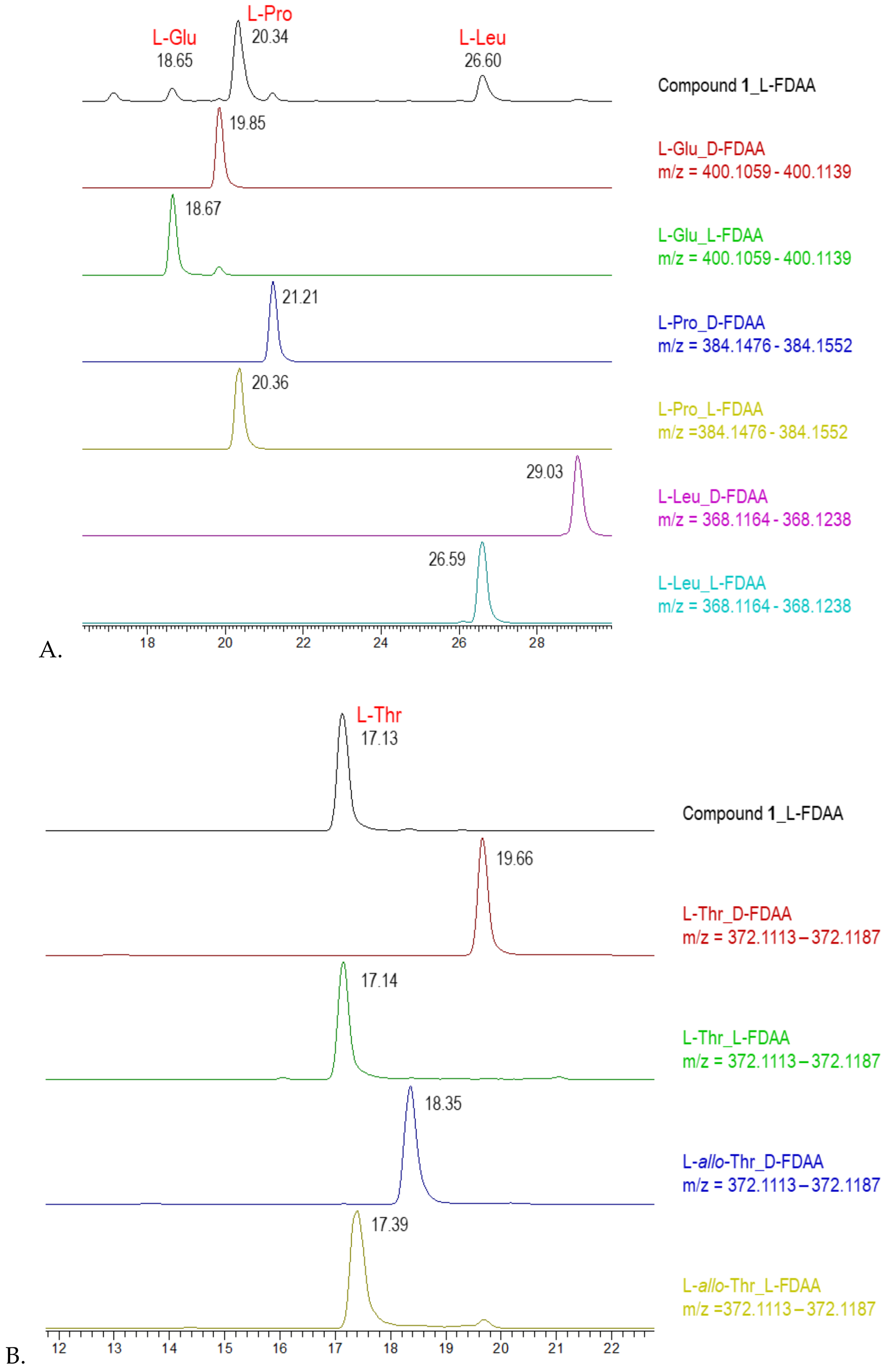

Figure S13. Advanced Marfey's analysis of compound **1**. Extracted-ion chromatograms at *m/z* 400.1099, at *m/z* 368.1201 and at *m/z* 384.1514 of the L-FDAA derivatives from the hydrolysis of **1** and of D- and L-FDAA derivatives L-Glu, L-Pro and L-Leu. (B) Extracted-ion chromatograms at *m/z* 372.1150 of the L-FDAA derivatives from the hydrolysis of **1** and of D- and L-FDAA derivatives L-Thr and L-*allo*-Thr.

Table S2. Full NMR data of compound **2** (^{1}H 600 MHz, ^{13}C 175 MHz, CD_3OD).

AA	pos.	δ_C, type		δ_H, mult (*J* in Hz)	HMBC
**pGlu**	NH				
	1	172.99, C			
	2	56.25, CH		4.56, dd (4.2, 8.8)	pGlu-5
	3	25.58, CH_2	a	2.50, m	pGlu-1, pGlu-5
			b	2.19, m	pGlu-1, pGlu-5
	4	30.38, CH_2	a	2.40, m	pGlu-5
			b	2.31, m	pGlu-5
	5	181.55, C			
**Pro^{I}**	1	174.5, C			
	2	61.37, CH		4.49, dd (4.3,8.5)	Pro^{I}-1
	3	30.36, CH_2	a	2.18, m	Pro^{I}-1
			b	1.97, m	Pro^{I}-1
	4	25.94, CH_2	a	2.03, m	
			b	1.98, m	
	5	48.06, CH_2	a	3.68, m	
			b	3.61, m	
**Leu**	NH				
	1	173.03, C			
	2	51.14, CH		4.62, t (7.2)	Leu-1, Pro^{I}-1
	3	41.12, CH_2		1.57, t (7.2)	Leu-1
	4	25.86, CH		1.81, nonet (6.6)	
	5	21.92, CH_3		0.96, d (6.6)	
**Pro^{II}**	1	172.70, C			
	2	59.67, CH		4.69 dd (4.5,8.3)	Leu-1, Pro^{II}-1
	3	29.19, CH_2	a	2.27, m	Pro^{II}-1
			b	1.98, m	Pro^{II}-1
	4	25.87, CH_2	a	2.13, m	
			b	2.01, m	
	5	48.62, CH_2	a	3.84, m	
			b	3.65, m	
**Pro^{III}**	1	175.46, C			
	2	60.35, CH		4.44, m	Pro^{II}-1,Pro^{III}-1
	3	30.08, CH_2	a	2.25, m	Pro^{III}-1
			b	2.00, m	Pro^{III}-1
	4	25.90, CH_2	a	2.07, m	
			b	2.04, m	
	5	48.50, CH_2	a	3.80, m	
			b	3.63, m	

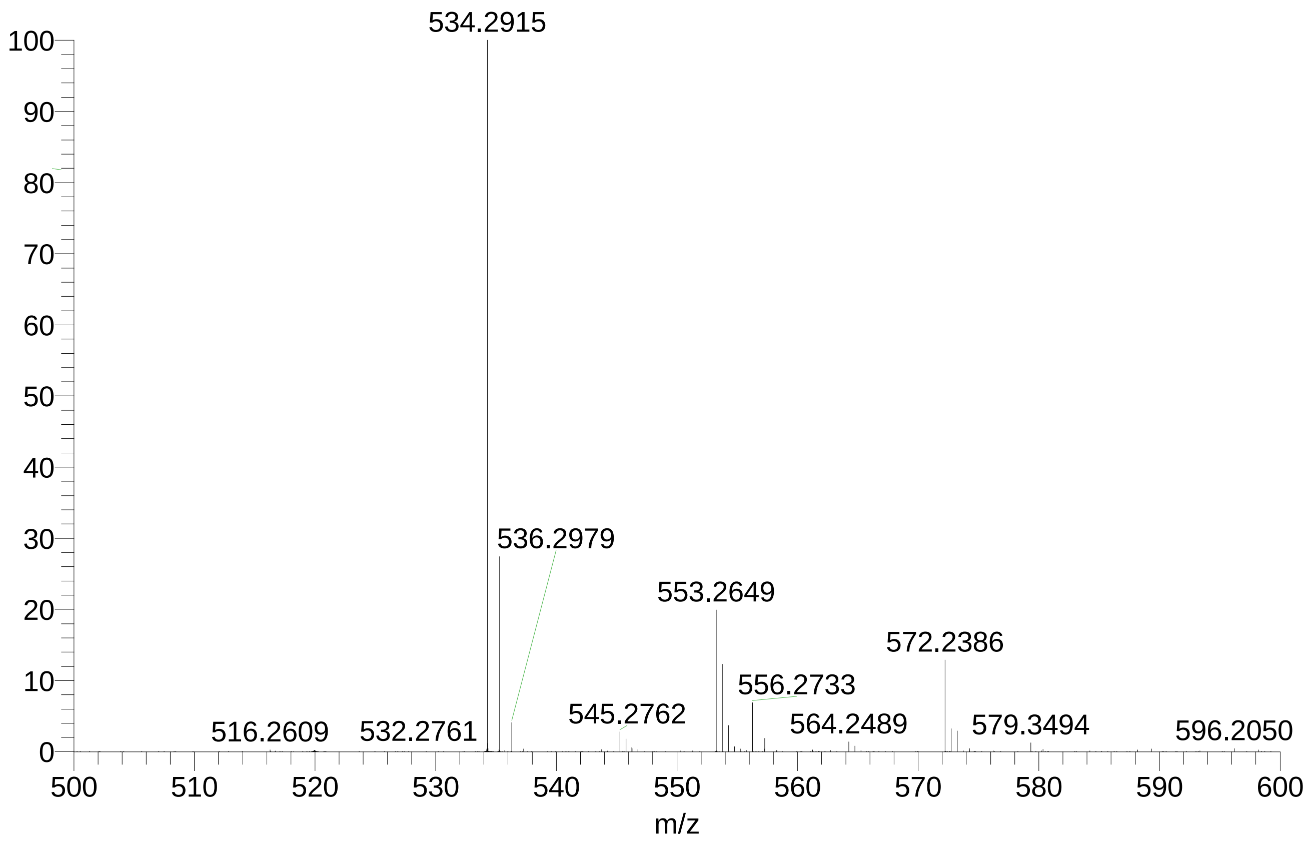

Figure S14. Positive ion mode HR-ESI mass spectrum of compound **2.**

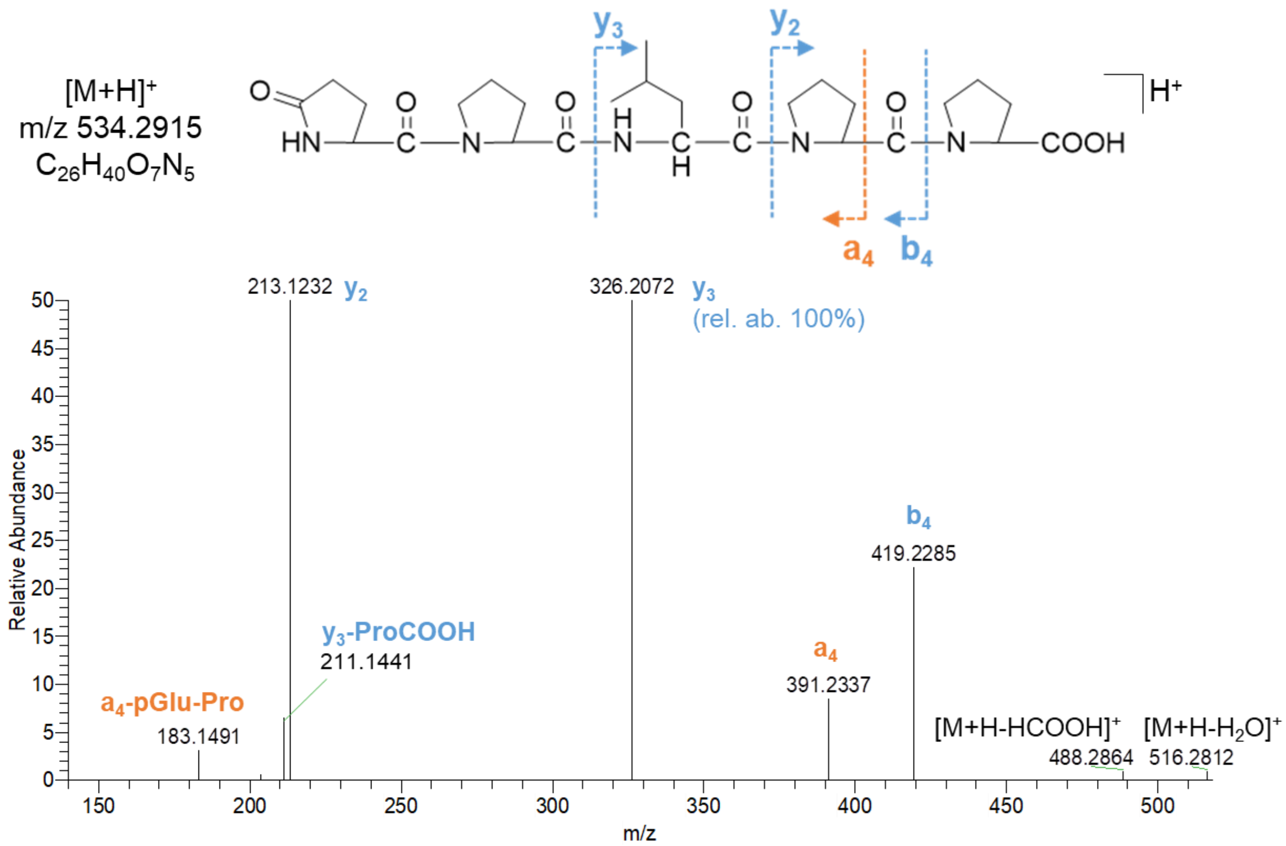

Figure S15. HR-MS/MS spectrum of the $[M+H]^+$ pseudomolecular ion of compound **2**.

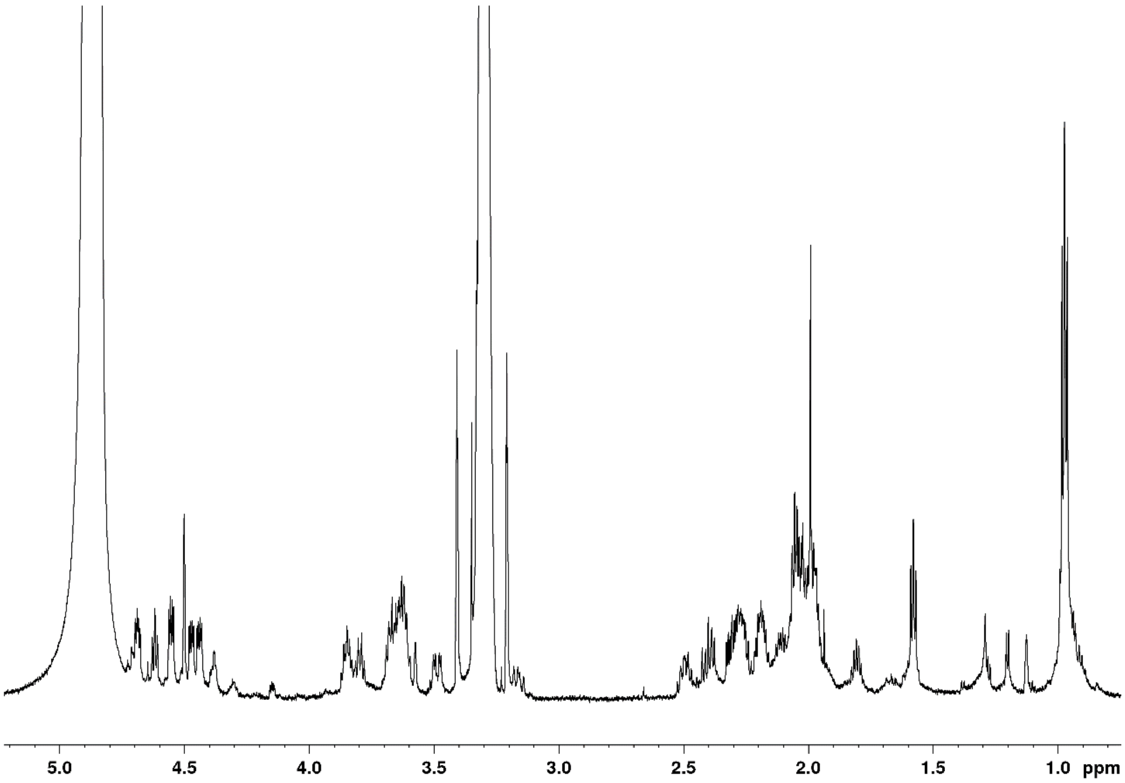

Figure S16. ^{1}H-NMR spectrum of compound **2** (700 MHz, CD_3OD).

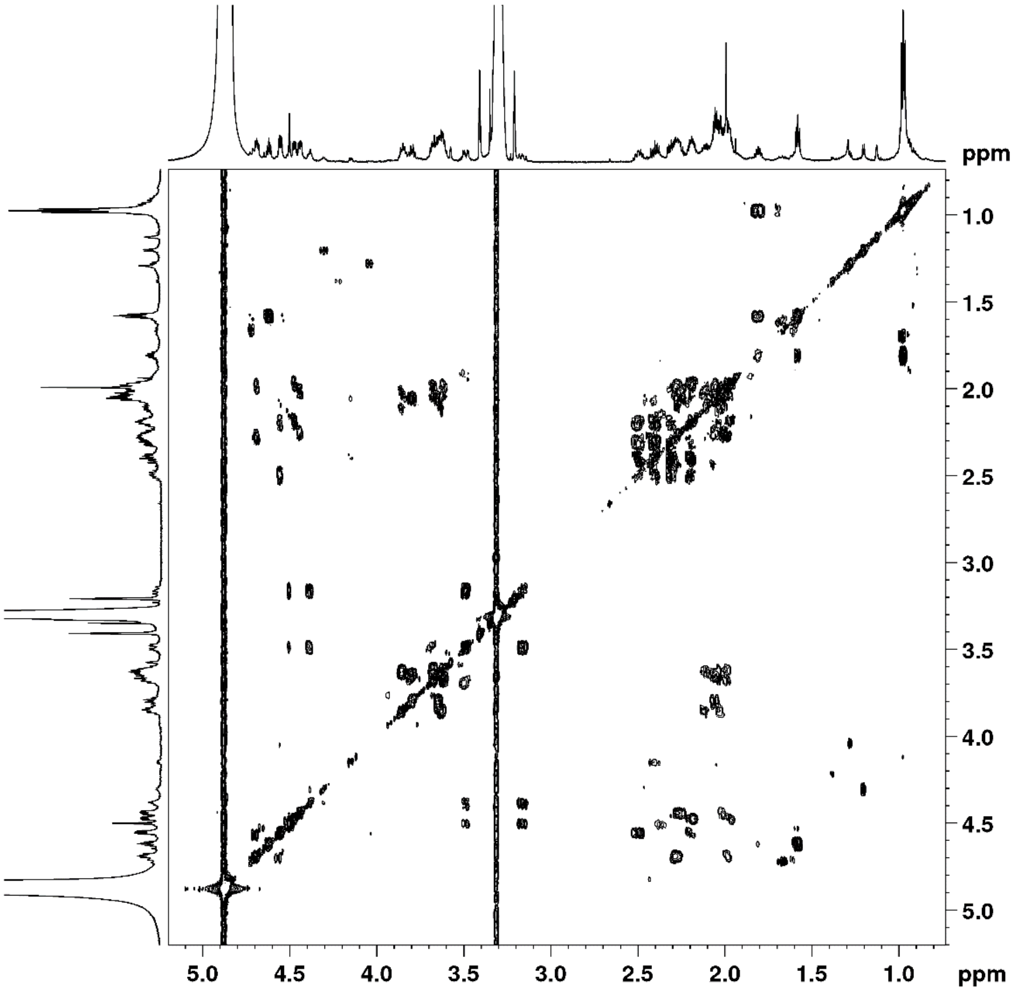

Figure S17. COSY spectrum of compound **2** (700 MHz, CD_3OD).

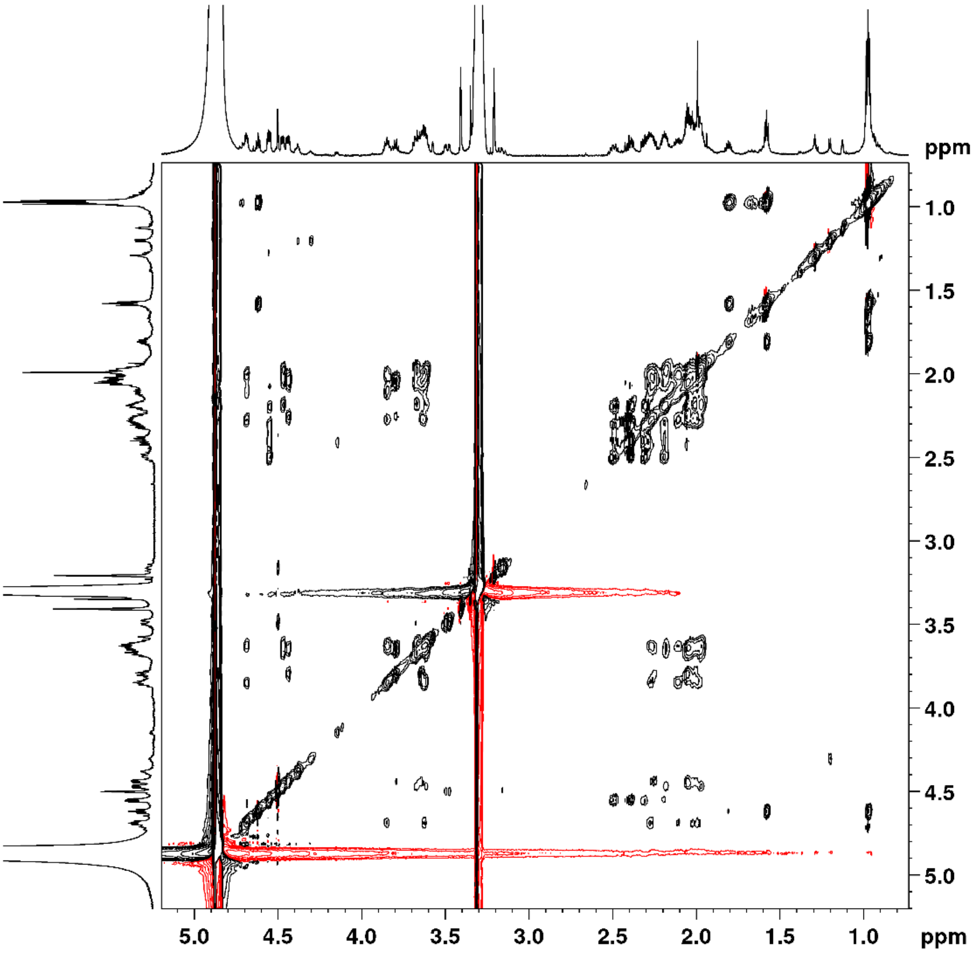

Figure S18. TOCSY spectrum of compound **2** (700 MHz, CD_3OD).

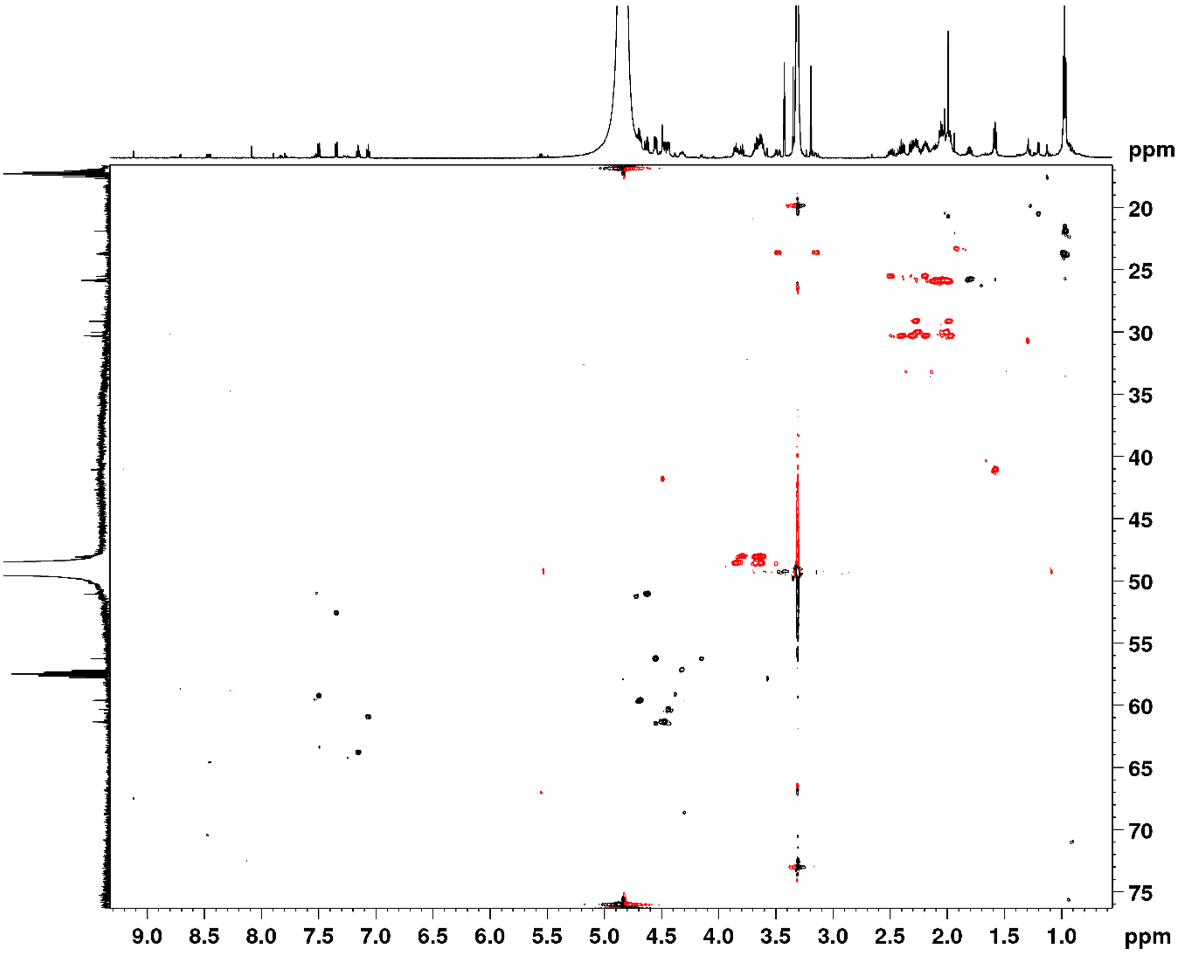

Figure S19. HSQC spectrum of compound **2** (600 MHz, CD_3OD).

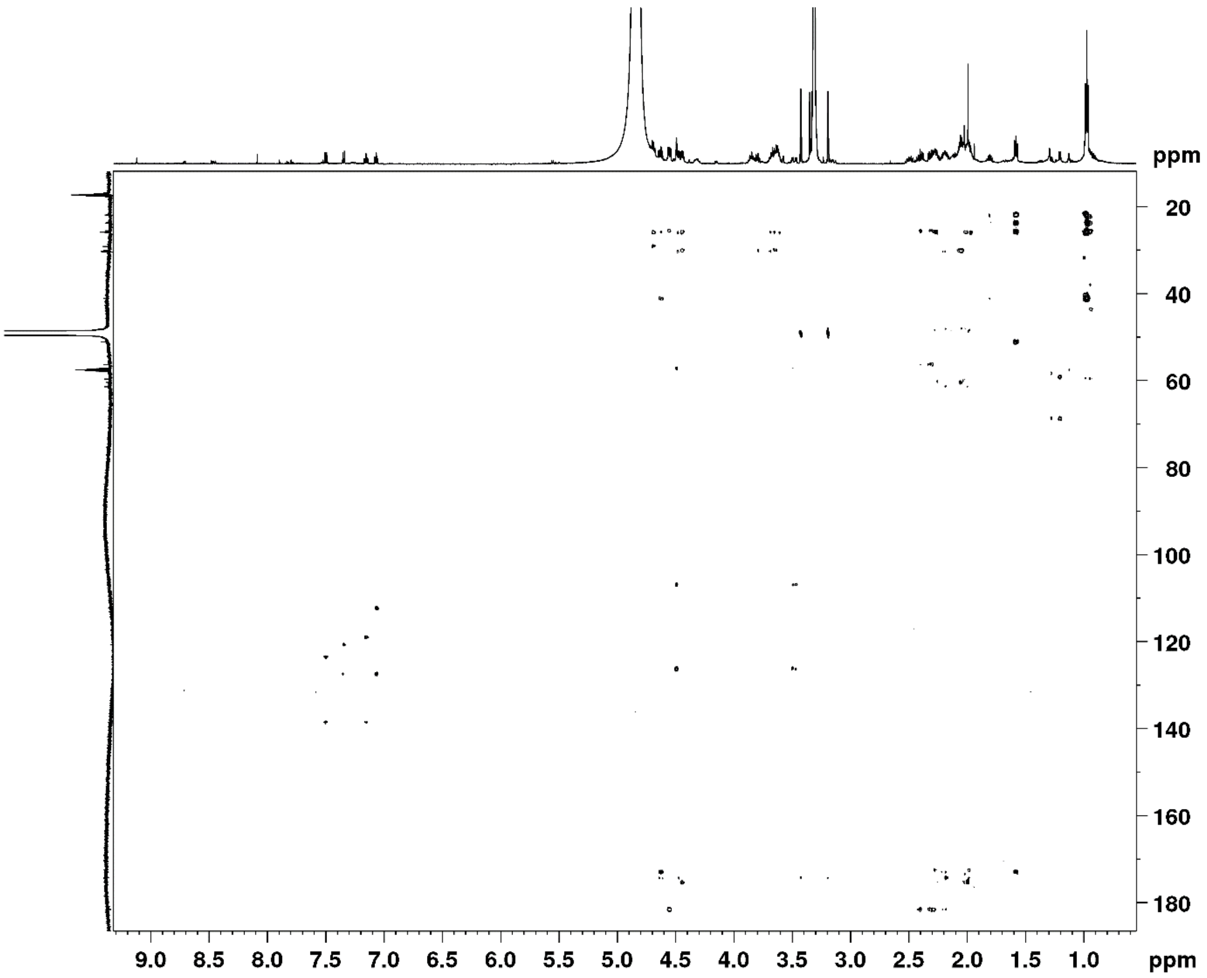

Figure S20. HMBC spectrum of compound **2** (600 MHz, CD_3OD).

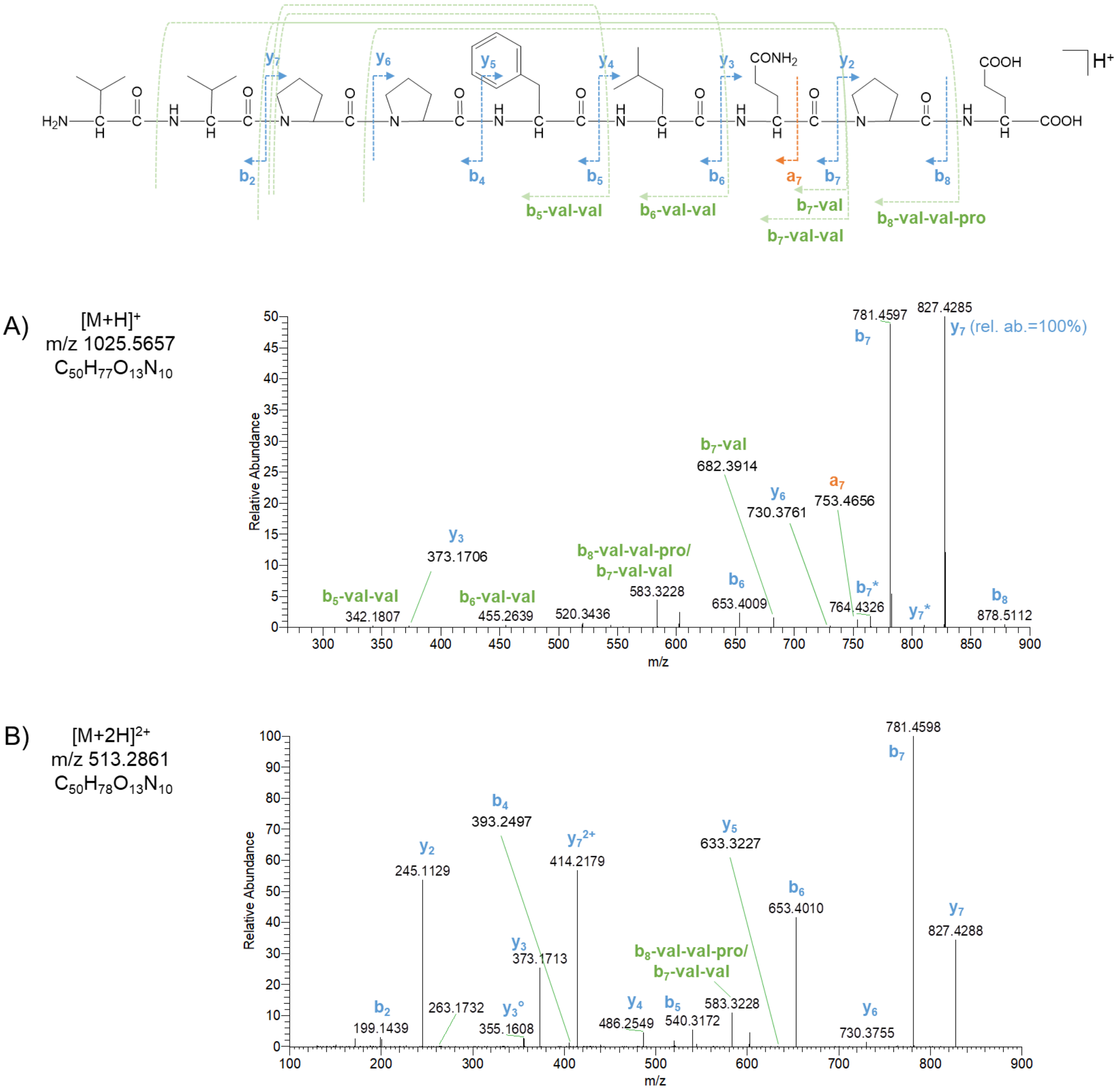

Figure S21. HR-MS/MS spectrum of the $[M+H]^+$ and $[M+2H]^{2+}$ ions of compound **3** (R_t = 17.3 min). Ions marked with a circle (°) derive from the corresponding fragment after water loss; ions marked with an asterisk (*) derive from the corresponding fragment after NH_3 loss.

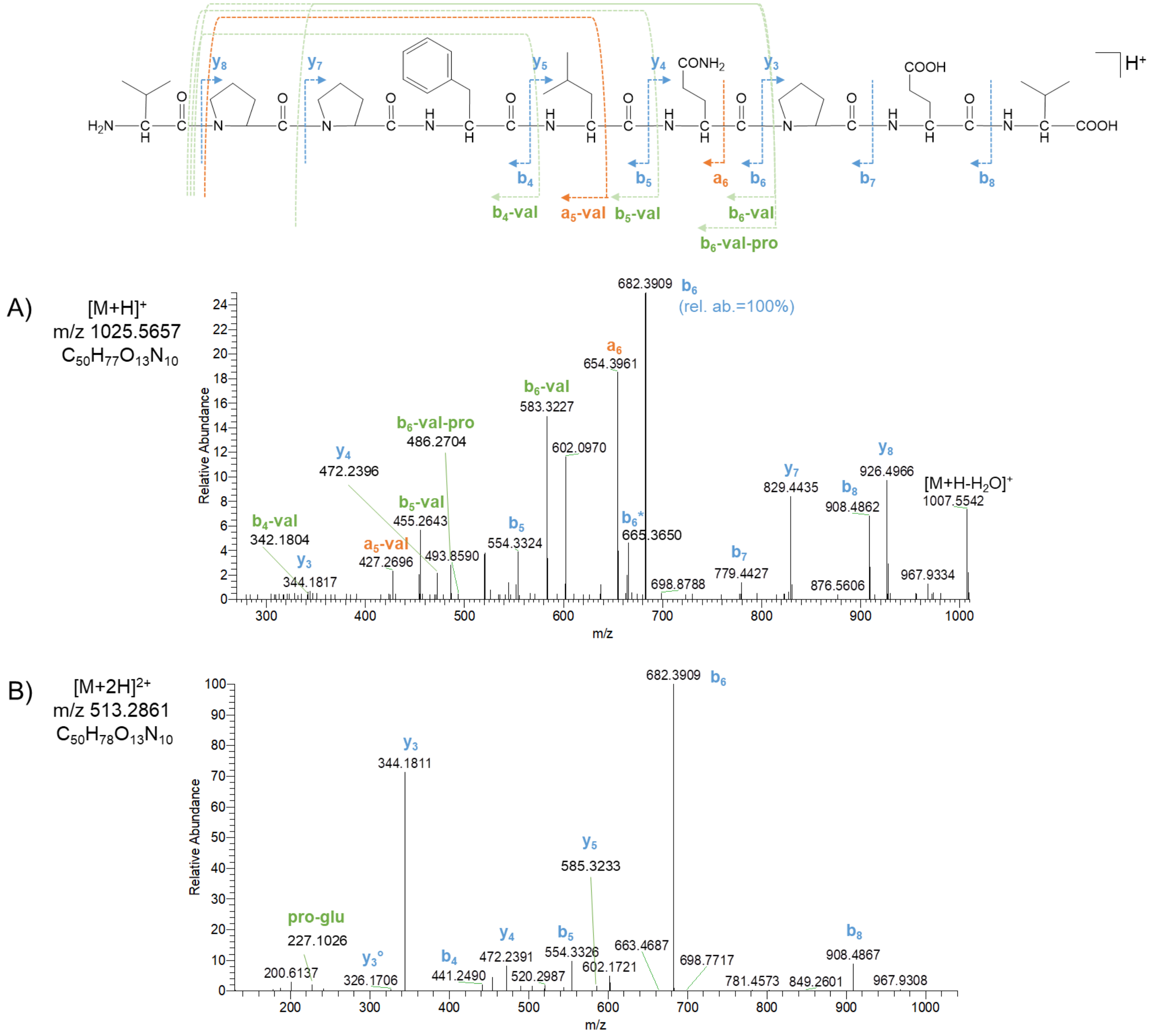

Figure S22. HR-MS/MS spectrum of the [M+H]+ and [M+2H]2+ ions of compound **4** (R_t = 18.4 min). Ions marked with a circle (°) derive from the corresponding fragment after water loss; ions marked with an asterisk (*) derive from the corresponding fragment after NH_3 loss.

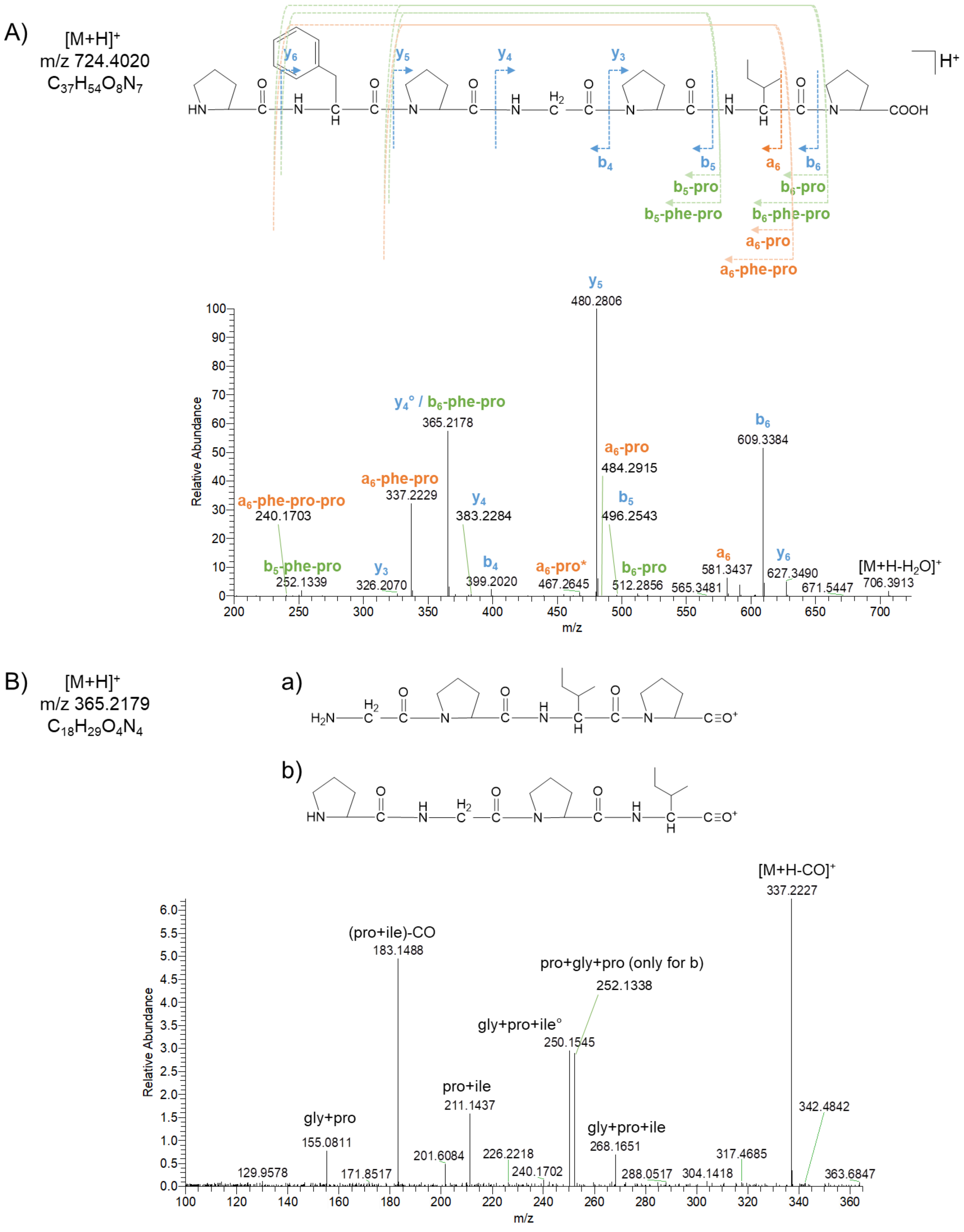
A)
[M+H]+
m/z 724.4020
C37H54O8N7
y6
y5
y4
y3
b4
b5
a6
b6
b5-pro
b6-pro
b5-phe-pro
b6-phe-pro
a6-pro
a6-phe-pro
H+
COOH
y5
480.2806
y4° / b6-phe-pro
365.2178
b6
609.3384
a6-pro
484.2915
a6-phe-pro
337.2229
y4
383.2284
b5
496.2543
a6-phe-pro-pro
240.1703
b5-phe-pro
252.1339
y3
326.2070
b4
399.2020
a6-pro*
467.2645
b6-pro
512.2856
565.3481
a6
581.3437
y6
627.3490
671.5447
[M+H-H2O]+
706.3913
Relative Abundance
m/z
B)
[M+H]+
m/z 365.2179
C18H29O4N4
a)
b)
C≡O+
[M+H-CO]+
337.2227
(pro+ile)-CO
183.1488
pro+gly+pro (only for b)
252.1338
gly+pro+ile°
250.1545
pro+ile
211.1437
gly+pro
155.0811
gly+pro+ile
268.1651
342.4842
129.9578
171.8517
201.6084
226.2218
240.1702
288.0517
304.1418
317.4685
363.6847
Relative Abundance
m/z

Figure S23. (A) HR-MS/MS spectrum of the $[M+H]^+$ pseudomolecular ion of compound **5** (R_t = 17.4 min). (**B**) HR-MS^3 spectrum of the fragment ions a) and b) at *m/z* 365.2179 arising from fragmentation of compound **5**. Ions marked with a circle (°) derive from the corresponding fragment after water loss; ions marked with an asterisk (*) derive from the corresponding fragment after NH_3 loss.

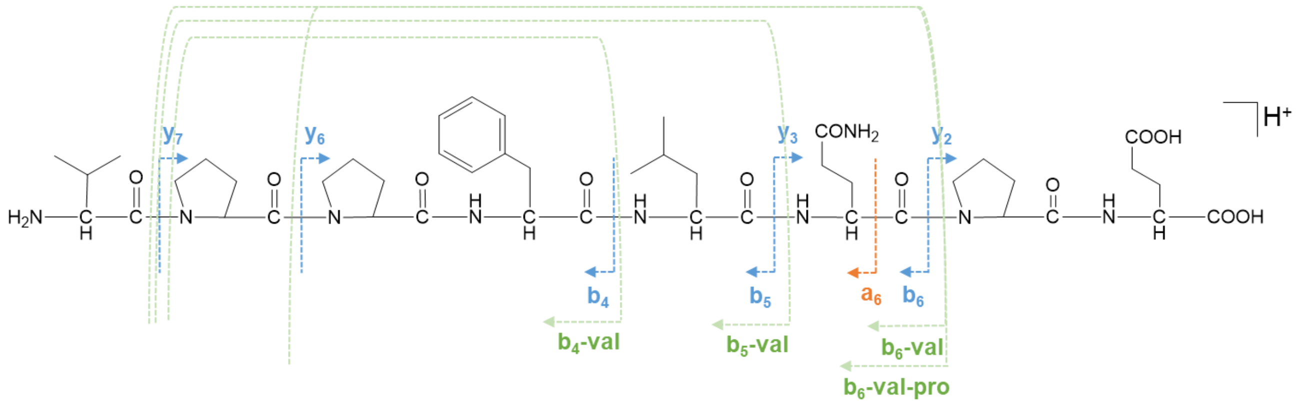

A)

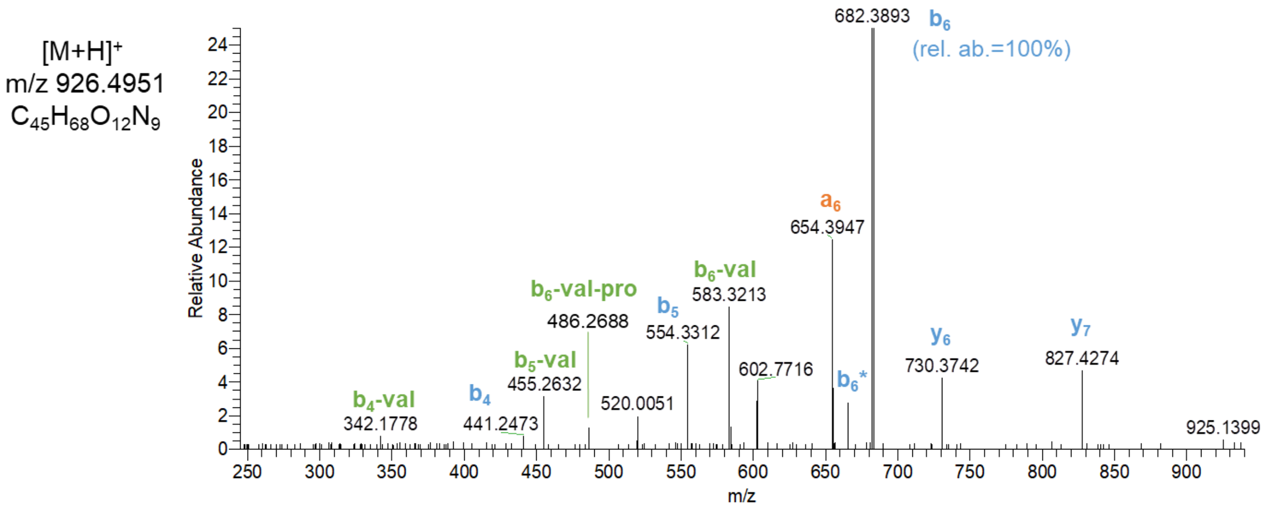

B)

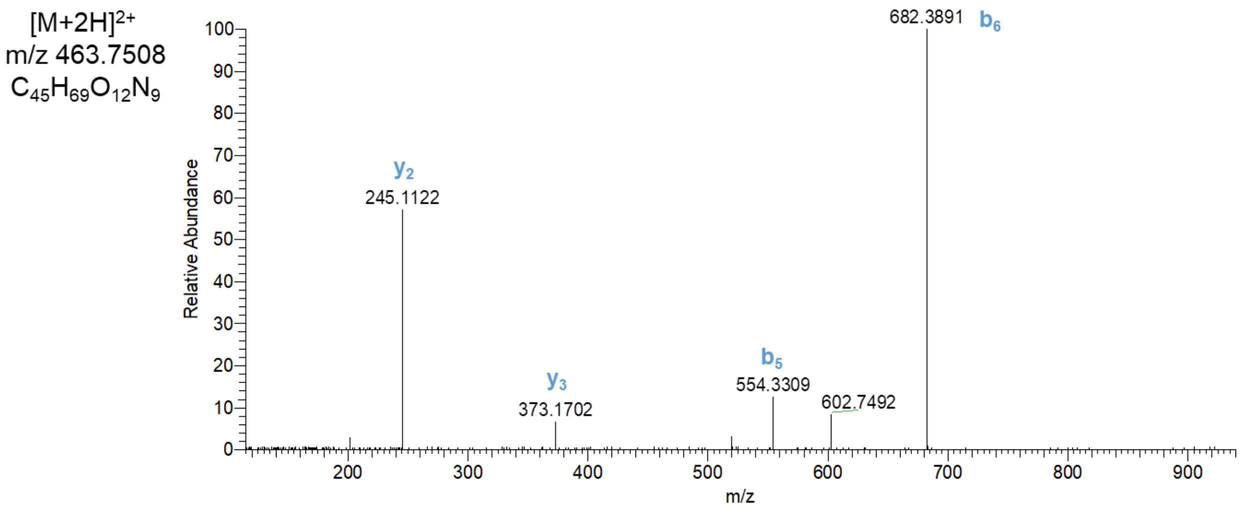

Figure S24. HR-MS/MS spectrum of the $[M+H]^+$ and $[M+2H]^{2+}$ ions of compound **6** (R_t = 16.0 min). Ions marked with a circle (°) derive from the corresponding fragment after water loss; ions marked with an asterisk (*) derive from the corresponding fragment after NH_3 loss.

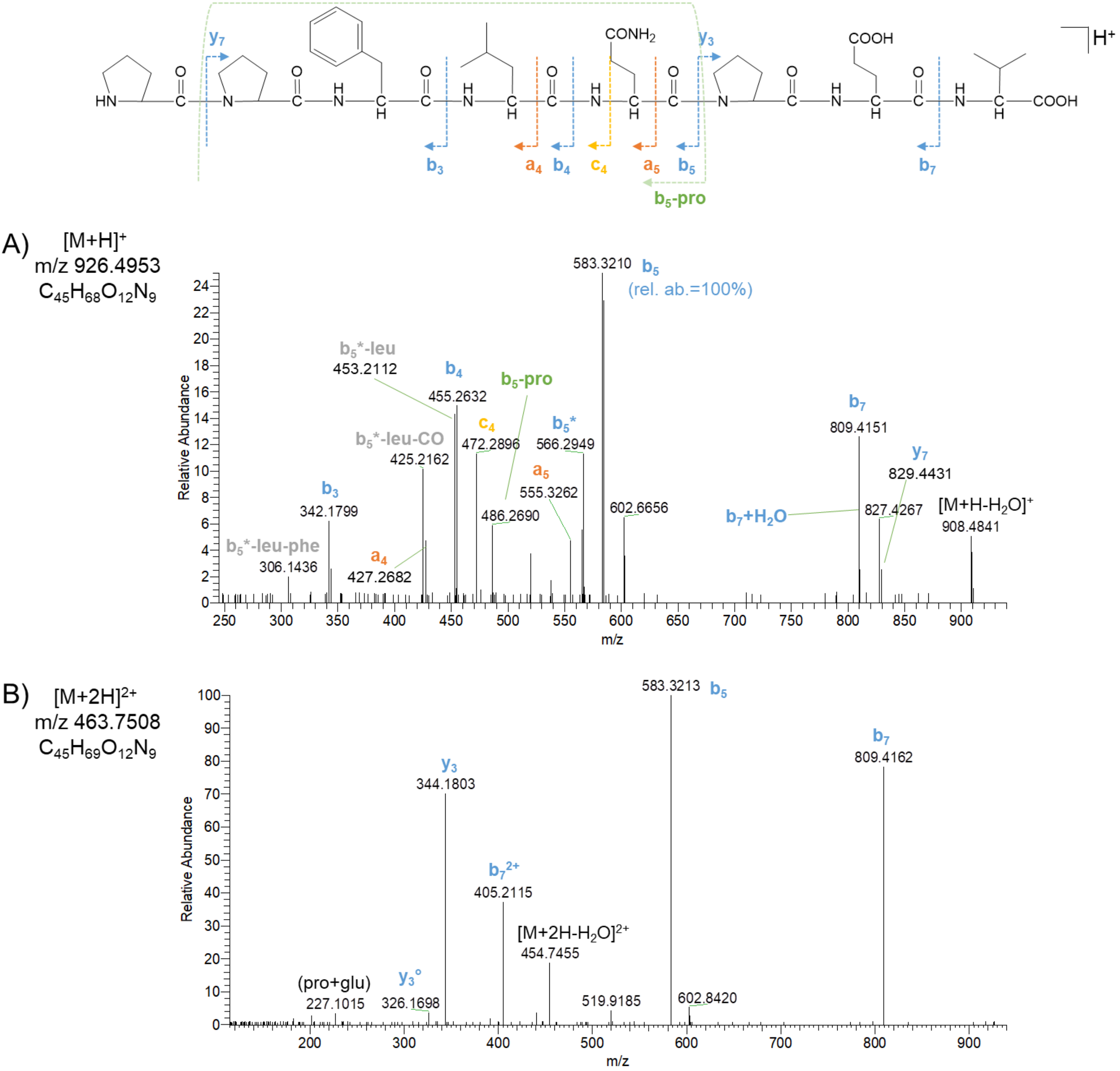

Figure S25. HR-MS/MS spectrum of the $[M+H]^+$ and $[M+2H]^{2+}$ ions of compound **7** (R_t = 17.0 min). The b_5* ion (**A**) is expected to form a macrocyclic intermediate, presumably deriving from head-to-side chain cyclization between the N-terminal Pro and the Gln side chain, with concomitant NH_3 loss. This intermediate functions as a precursor to the formation of non-native, or scrambled, product ions (highlighted in grey). Ions marked with a circle (°) derive from the

corresponding fragment after water loss; ions marked with an asterisk (*) derive from the corresponding fragment after NH_3 loss.

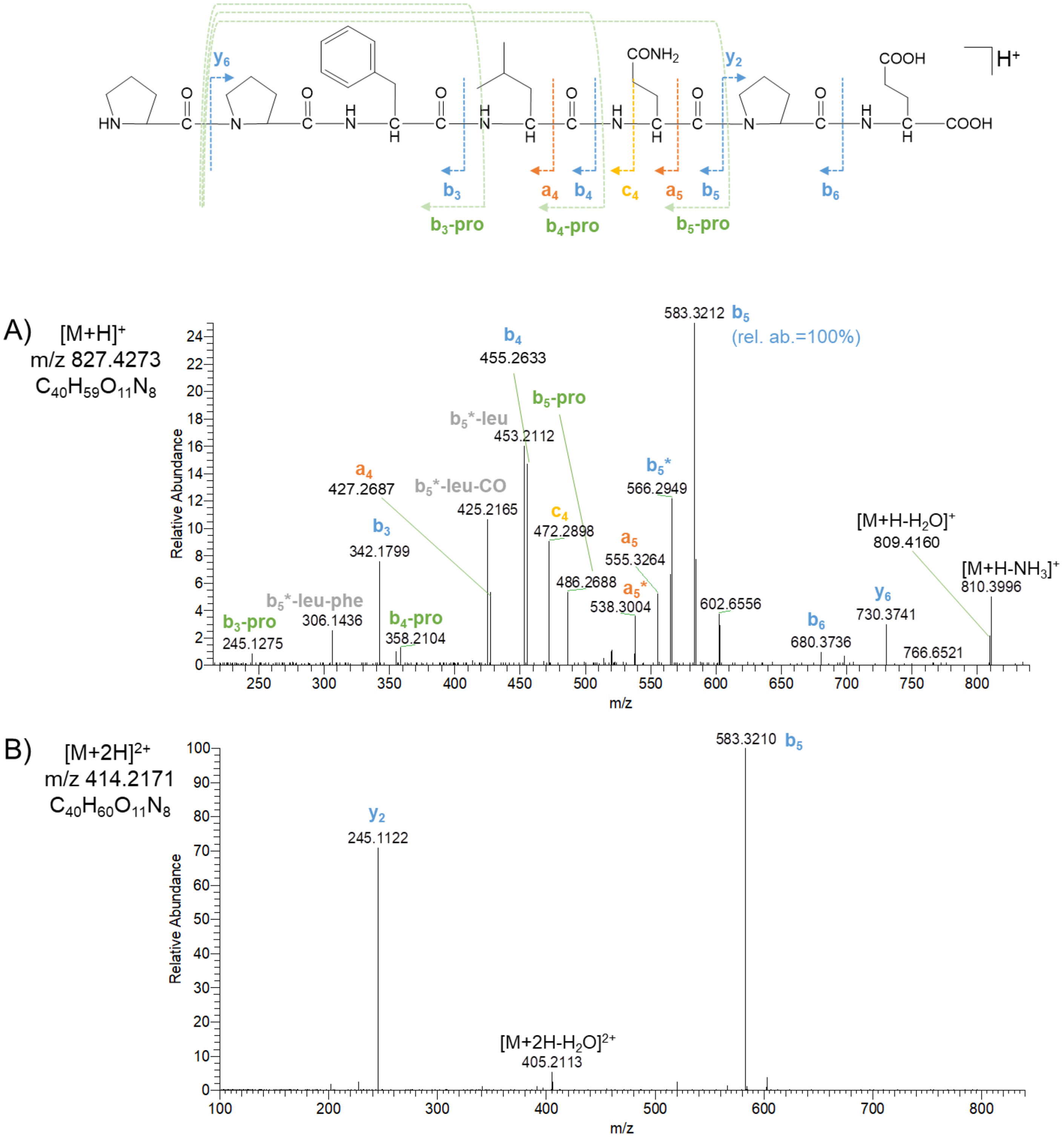

Figure S26. HR-MS/MS spectrum of the $[M+H]^+$ and $[M+2H]^{2+}$ ions of compound **8** (R_t = 14.2 min). The b_5* ion (**A**) is expected to form a macrocyclic intermediate, presumably deriving from head-to-side chain cyclization between the N-

terminal Pro and the Gln side chain, with concomitant NH_3 loss. This intermediate functions as a precursor to the formation of non-native, or scrambled, product ions (highlighted in grey). Ions marked with a circle (°) derive from the corresponding fragment after water loss; ions marked with an asterisk (*) derive from the corresponding fragment after NH_3 loss.

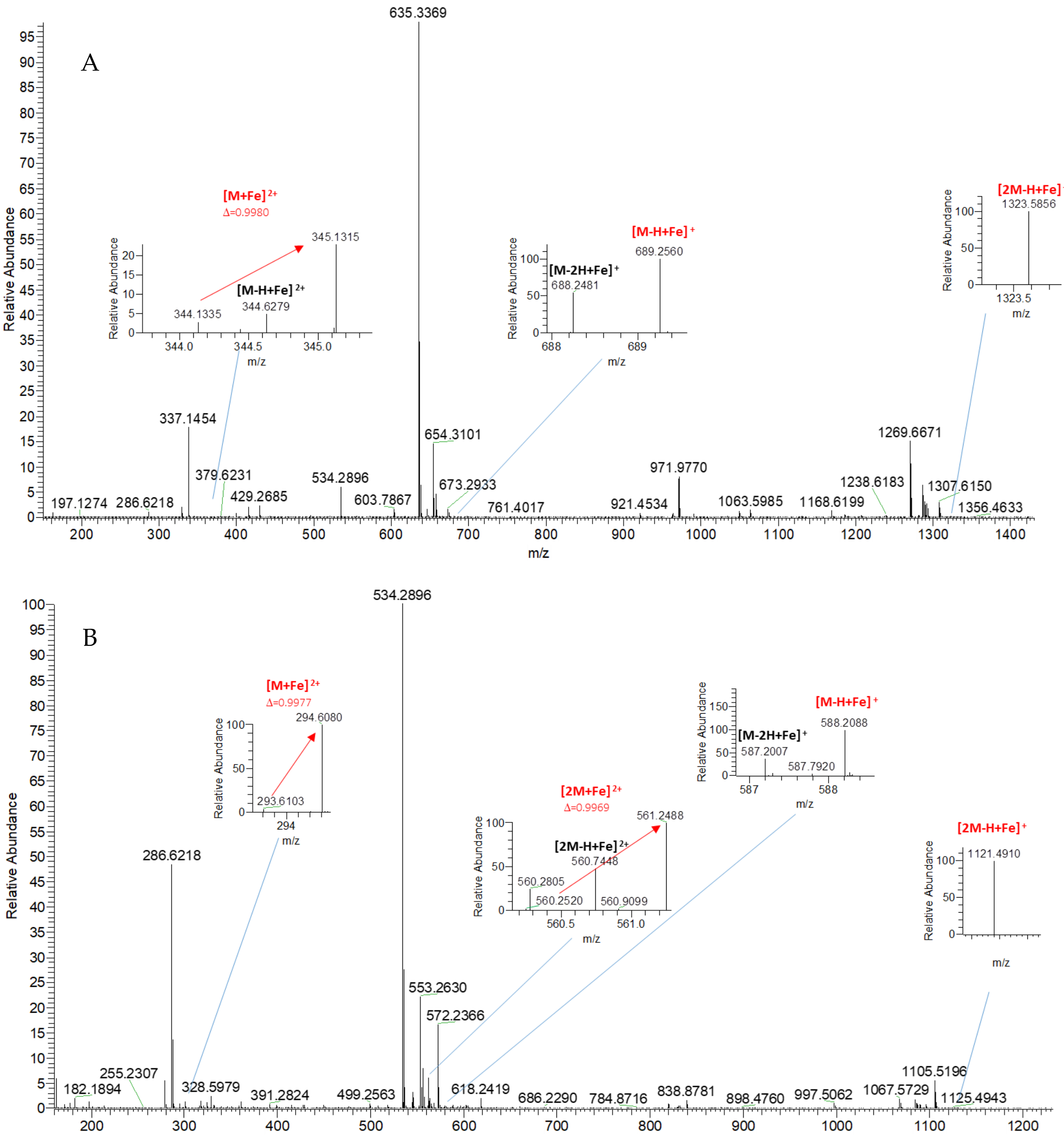

Figure S27. HRMS spectra showing iron adducts of compounds **1** (A) and **2** (B). Iron (II) adducts are depicted in red while iron (III) adducts in black. Where discernible, the typical mass difference between the two observable iron isotopes ^{56}Fe and ^{54}Fe was highlighted.

Table S3. Ferric reducing antioxidant power assay results of compounds **1** and **2**.[a]

	compound **1**	compound **2**	Ascorbic acid
Abs 4 min (nm)	0.04	0.08	0.2
a.a. equivalents (μM)	21 ± 0	40 ± 1	-
FRAP value	41 ± 1	80 ± 2	-
Abs 16 min (nm)	0.05	0.12	0.2
a.a. equivalents (μM)	26 ± 0	60 ± 2	-
FRAP value	52 ± 2	121 ± 4	-

[a] The samples and controls were analysed at 593 nm in triplicates in order to infer the standard deviation, and the absorbance values reported were corrected for the blank reading (0.07). The results were compared with ascorbic acid 100 μM in order to calculate Ascorbic Acid (a.a.) equivalents values and in turn the FRAP values.

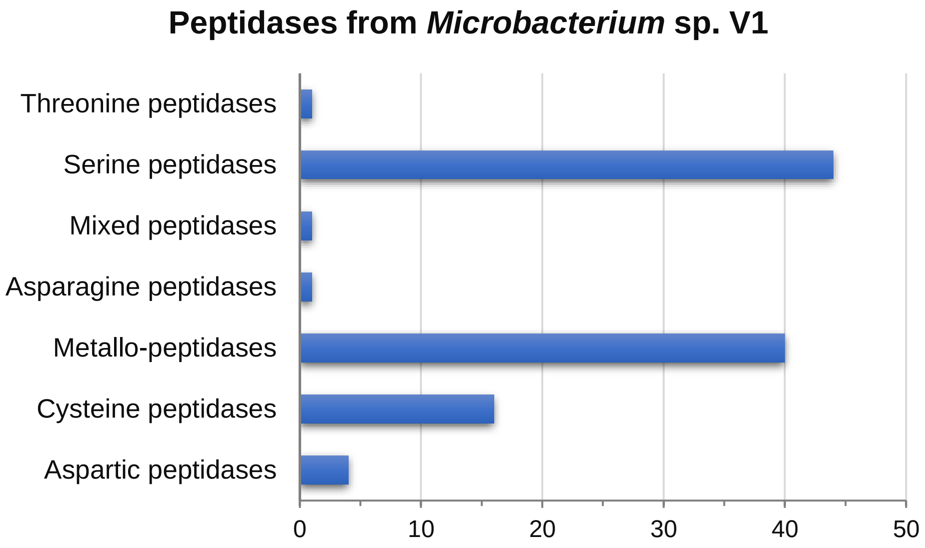

Figure S28. Peptidases from *Microbacterium sp.* V1 annotated by using the bioinformatic tool Hotpep-protease.
